# Stromal FAP^+^ cancer associated fibroblasts orchestrate a pro‐tumorigenic niche with malignant proliferative stemness and cancer progression

**DOI:** 10.1002/ctm2.70688

**Published:** 2026-05-13

**Authors:** Jiajin Wu, Bingxin Liu, Silu Chen, Junyi Xin, Meilin Wang

**Affiliations:** ^1^ Key Laboratory of Environmental Medicine Engineering, Ministry of Education of China, School of Public Health Southeast University Nanjing China; ^2^ Jiangsu Key Laboratory of Innovative Cancer Diagnosis & Therapeutics, Jiangsu Cancer Hospital, Jiangsu Institute of Cancer Research The Affiliated Cancer Hospital of Nanjing Medical University Nanjing China

1

The remodelling of the tumour microenvironment (TME) plays a pivotal role in driving the malignant progression of bladder cancer.^1^, ^2^ However, the dynamic molecular determinants, and regulatory mechanisms governing intercellular communication during bladder cancer progression remain poorly defined.^3^ Here, by integrating single‐cell spatiotemporal multi‐omics analysis and functional experiments validation, we identified a tumour–stroma interactive niche composed of FAP^+^ cancer associated fibroblasts (CAFs) and malignant tumour cells harbouring activation of the Malignant Proliferative Stemness Metaprogram (MPS‐MP), which collectively promotes the malignant progression of bladder cancer.

In this study, we integrated Nanjing Bladder Cancer (NJBC) scRNA‐seq datasets with public resources to construct dynamic cellular ecosystems underlying bladder cancer progression (Figure 1A,B, Table S1, and Table S2). Following stringent quality control, 633 702 high‐quality cells were retained (Figure 1C, and Figure S1A–D). Unsupervised clustering identified nine major cell lineages, including epithelial, T, myeloid, fibroblast, endothelial, B, plasma, mast and neural cells (Figure 1D, and Figure S2A–C). Notably, epithelial cell abundance was substantially elevated in muscle‐invasive bladder cancer (MIBC) patients, revealing progressive and systemic TME remodelling during disease advancement (Figure 1E,G).

**FIGURE 1:**
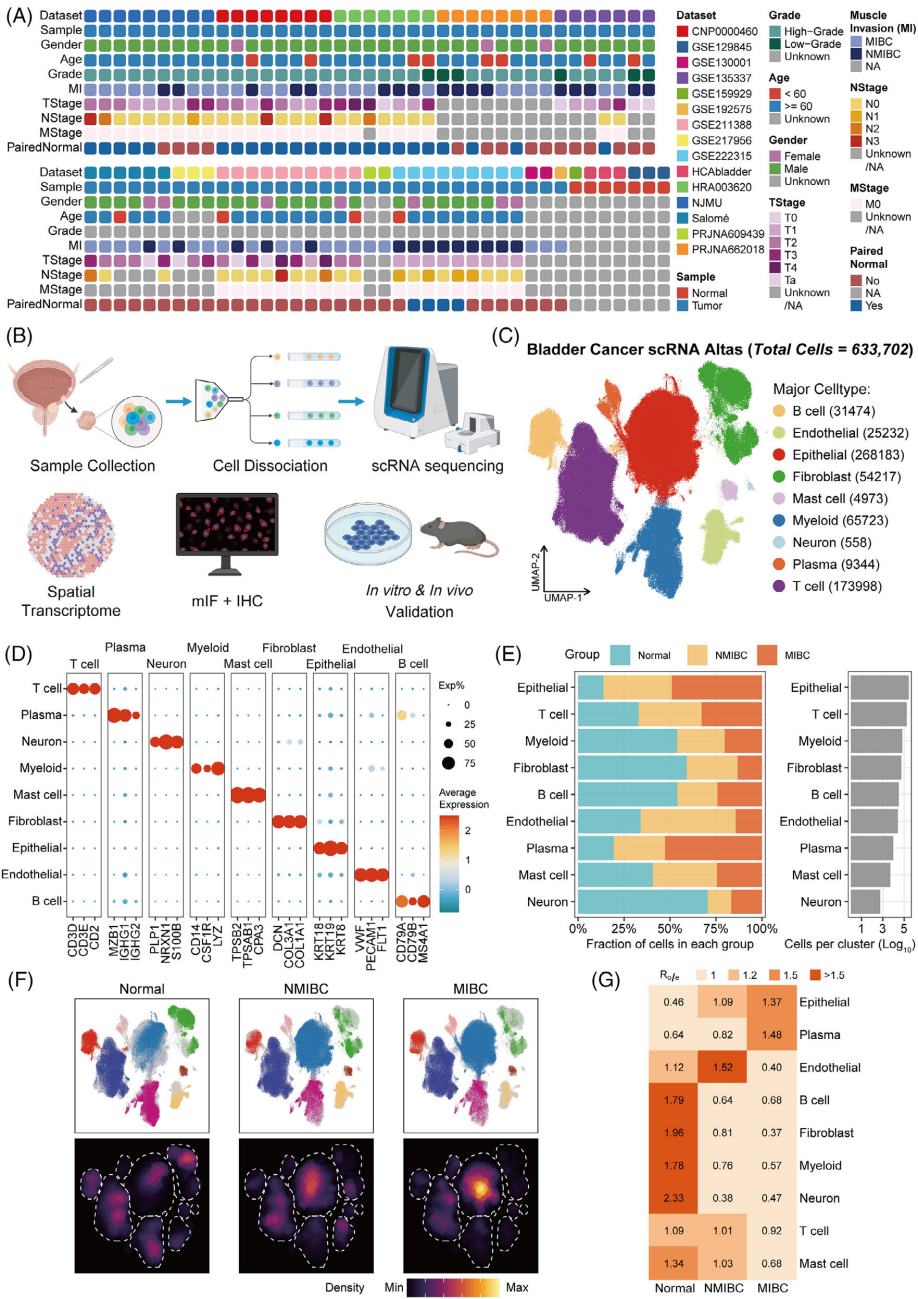
Dynamic characterization of single‐cell bladder cancer atlas. (A) Heatmap summarizing the clinicopathological and technical metadata of all specimens. (B) Schematic overview of the study workflow. (C) UMAP embedding of the bladder cancer scRNA atlas. (D) Dot plot showing the canonical marker genes used for lineage annotation across major cell populations. (E) Stacked bar plots depicting the relative cellular composition of each major lineage across normal bladder tissue, Non–Muscle‐Invasive Bladder Cancer (NMIBC) and MIBC. (F) Group‐specific UMAP distributions and corresponding density maps. (G) Heatmap of the Ro/e for each major cell lineage across disease states.

To pinpoint the cellular basis of genetic susceptibility within this remodelled ecosystem, we mapped disease risk score to the single‐cell level^4^ (Figure S3A,B). High‐risk trait scores were predominantly enriched in epithelial cells and fibroblasts, with a more pronounced signal observed in MIBC samples (Figure 2A). Combination annotation of epithelial cells using “InferCNV” and “Cancer‐Finder” algorithms identified 199 912 malignant tumour cells (Figure S4A–D). cNMF‐based decomposition of malignant cells further delineated 11 transcriptional meta‐programs, covering ribosomal translation, cell cycle, oxidative stress, malignant proliferative stemness, cell adhesion, metabolic reprogramming, luminal differentiation, epithelial‐mesenchymal transition (EMT)/Claudin‐related programs, immune response and neuroendocrine differentiation (Figure 2B, Figures S5A,B and S6). Among these, MPS‐MP and essential marker genes (ATF3, CXCL8, EFNA1 and LAMC2) were significantly enriched in MIBC samples (Figure 2D, Figures S7A,B and S9A, and Table S3). Consistently, “DUBStepR”‐based clustering further demonstrated that MIBC‐specific malignant clusters exhibited elevated MPS‐MP activated cells and up‐regulated essential marker genes (Figure 2C,E, Figure S8A‐D and S9B,C). We performed transcription factor enrichment analysis using “decoupleR” and identified ATF3 as a key driver of the MPS‐MP program, regulating the expression of critical downstream target genes, including CXCL8, MAPK8, EPAS1, EGR1 and VEGFA (Figure S10A). Gene set enrichment analysis of MPS‐MP‐high malignant cells revealed strong enrichment of TGF‐β signalling, EMT, and hypoxia Hallmark pathways, supporting the central role of MPS‐MP in driving the aggressive cellular state during bladder cancer progression (Figure 2F, and Figure S11). Furthermore, we conducted cell–cell communication analysis between MPS‐MP and other cells among tumour microenvironment. Compared with MPS‐MP inactive cells, MPS‐MP activated tumour cells exhibited a markedly increased number and strength of interactions with stromal and immune cell populations (Figure S12A,B).

**FIGURE 2:**
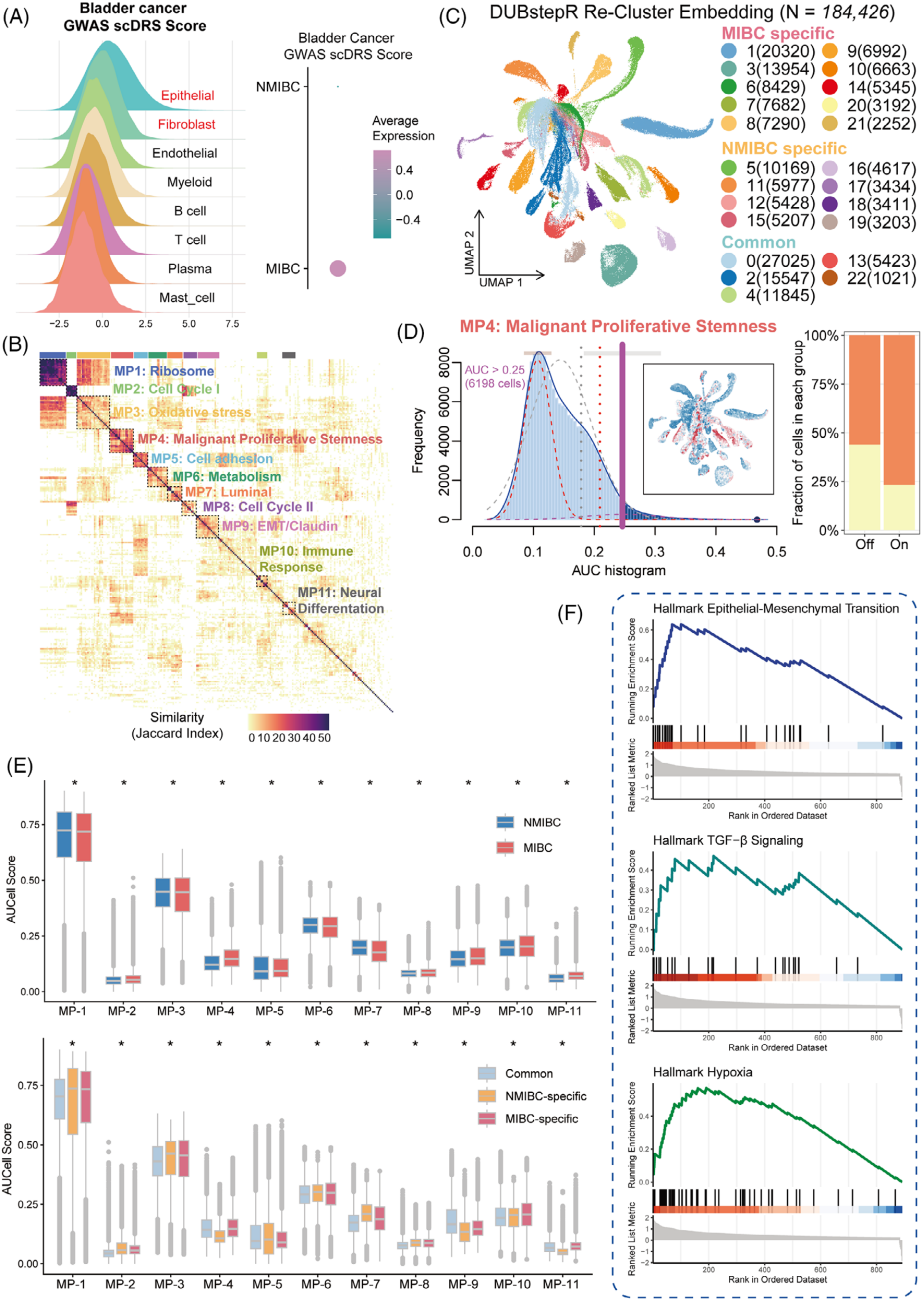
Identification of a malignant proliferative‐stemness meta‐program associated with bladder cancer progression. (A) Ridge plots showing the distribution of bladder cancer scDRS score across major cellular compartments. (B) Heatmap of malignant epithelial transcriptional programs, revealing 11 recurrent meta‐programs. (C) “DUBStepR”‐based re‐clustering of epithelial cells identifies 23 transcriptionally distinct subclusters on the UMAP embedding. (D) MPS‐MP active cells are preferentially enriched in MIBC compared with NMIBC. (E) Boxplots of enrichment scores for the 11 meta‐programs across NMIBC and MIBC samples and “DUBStepR” subclusters. (F) Gene set enrichment analysis of the MPS‐MP program. *: *p* < 0.05.

We next assessed links between major TME populations and clinical outcome using integrative deep‐learning modelling with the “scSurv” framework.^5^ We identified fibroblasts as the dominant risk contributors, implicating them as critical drivers of progression and poor survival (Figure 3A and Figure S13A–C). Consistently, fibroblasts exhibited the highest prognostic risk scores (Figure 3B and Figure S14A–C). Supervised selection using “Scissor” algorithm further showed that Scissor^+^ poor prognosis‐related cells were predominantly enriched within the fibroblast lineage^6^ (Figure 3C and Figure S15A–C). In parallel, malignant phenotype scoring based on “CancerSEA” and “Hallmark” signatures revealed marked enrichment of invasion, metastasis, hypoxia and proliferation phenotypes in fibroblasts (Figure 3D). Bulk RNA‐seq deconvolution across TCGA and GEO bladder cancer cohorts further demonstrated that fibroblast abundance scores derived from EPIC, ESTIMATE and MCPcounter algorithms were significantly associated with poor survival outcomes (Figure S16A,B), nominating CAFs as a key independent risk factor in the progression of bladder cancer. We also performed comparative cell communication analysis using “CellChat”, and found that tumour tissues displayed a higher number and greater strength of inferred interactions^7^ (Figure 3E). Differential network and information‐flow analyses revealed pronounced amplification of fibroblast‐centred signalling, with especially prominent interactions between fibroblasts and epithelial (Figure S17A,B). Notably, multiple pathways linked to stromal remodelling, adhesion and migration, immune regulation and angiogenesis exhibited elevated information flow in tumours, indicating that CAF‐driven communication circuits represent a major component of aberrant TME activation (Figure S17C).

**FIGURE 3:**
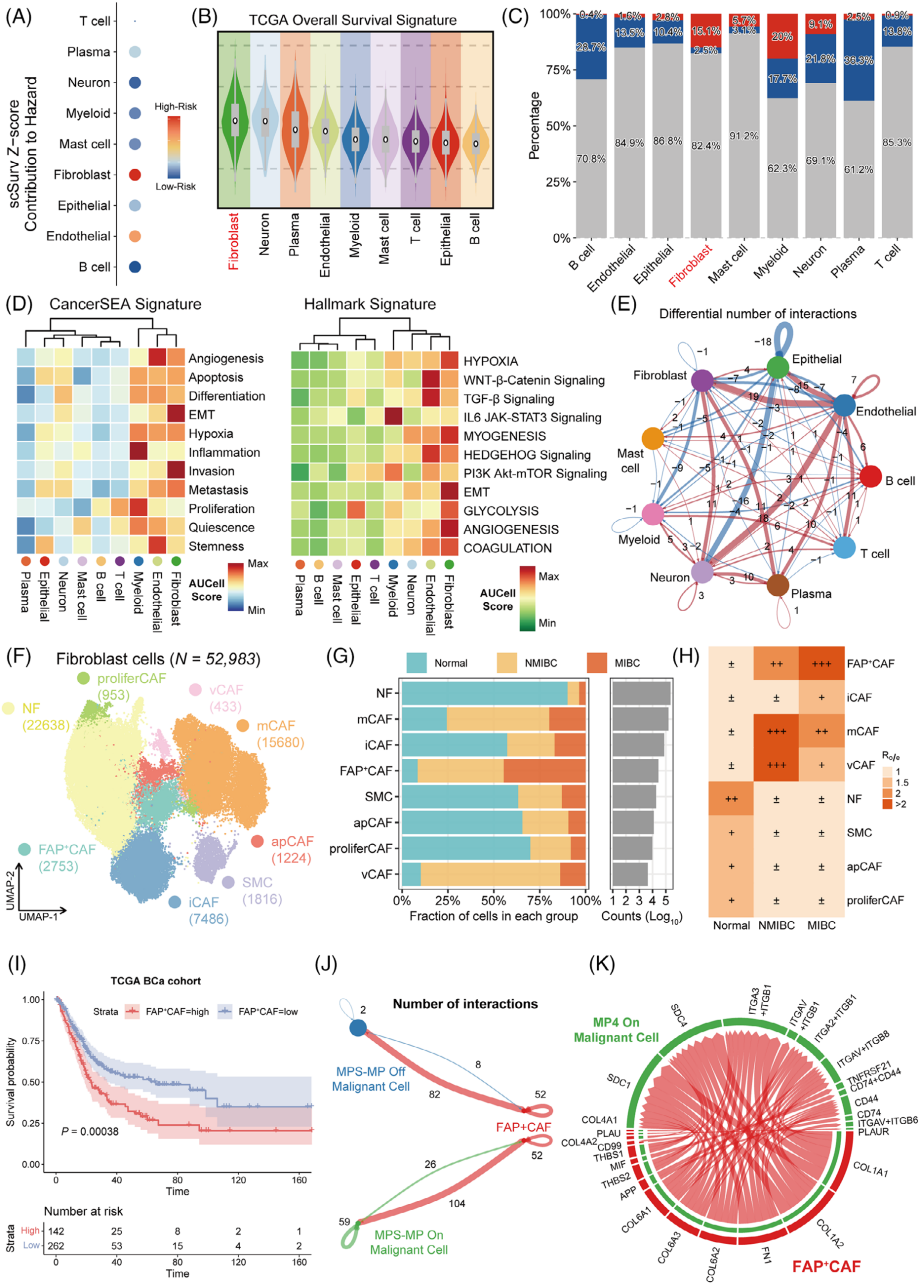
FAP^+^ CAFs represent the predominant high‐risk stromal compartment, and poor survival outcome in bladder cancer. (A) Cell type resolved scSurv analysis showing the Z‐score contribution of each major lineage to hazard. (B) Violin plots of the TCGA OS signature across major cell populations. (C) Stacked bar plots showing the distribution of scSurv defined risk states across major cell lineages. (D) Heatmaps of pathway enrichment scores for “CancerSEA” and Hallmark signatures across major cell populations. (E) Differential cell–cell communication network inferred from ligand–receptor analysis. (F) UMAP embedding of fibroblasts identifies eight transcriptionally distinct fibroblast subsets. (G) Stacked bar plots showing the relative distribution of fibroblast subsets across normal bladder tissue, NMIBC and MIBC samples. (H) Heatmap showing the Ro/e index of each fibroblast subset across disease states. (I) Kaplan–Meier survival analyses in the TCGA bladder cancer cohort stratified by FAP^+^ CAFs enrichment level. (J) Interaction network summarizing ligand‐receptor communication between FAP^+^ CAFs and malignant epithelial cells with MPS‐MP Off or MPS‐MP On states. (K) Chord diagram illustrating key ligand‐receptor interactions between FAP^+^ CAFs and MPS‐MP On malignant cells.

Having established fibroblasts as central risk mediators in bladder cancer, we next resolved fibroblast heterogeneity within the single‐cell atlas. Dimensionality reduction and clustering identified 11 fibroblast clusters (Figure S18A,B). After clustering and annotation, a total of 52 983 fibroblasts were classified into eight subsets based on canonical marker genes: normal fibroblast (NF), myofibroblast (mCAF), inflammatory cancer‐associated fibroblast (iCAF), fibroblast activation protein–positive cancer‐associated fibroblast (FAP^+^ CAF), antigen‐presenting cancer‐associated fibroblast (apCAF), proliferating cancer‐associated fibroblast (proliferCAF), vascular cancer‐associated fibroblast (vCAF) and smooth muscle cell (SMC) populations^8^ (Figure 3F, and Figure S18C). Cell preference analysis demonstrated a significant enrichment of FAP^+^ CAF in MIBC (Figure 3G,H and Figure S19A–D), implicating this subset as a major pro‐tumorigenic population during bladder cancer progression. To directly link CAF heterogeneity with clinical outcome, we derived prognosis‐related signatures and performed functional enrichment analysis. FAP^+^ CAF displayed consistently elevated risk scores for OS, DSS and PFI (Figure S20A–C), supporting a pivotal role in driving tumour progression and survival risk. We further identified poor prognosis‐associated cells that were primarily concentrated within the FAP^+^ CAF subset (Figure S21A–C). At the functional level, FAP^+^ CAF showed stronger enrichment of EMT and glycolytic programs (Figure S22A,B). Collectively, these findings indicate that FAP^+^ CAF subpopulations exhibit invasive phenotype acquisition, hypoxic responses, and angiogenesis. Using “BayesPrism” to deconvolute bulk RNA‐seq data, we found that patients with high FAP^+^ CAF abundance had significantly worse OS in both TCGA and GEO cohorts^9^ (Log‐rank *p* < 0.001; Figure 3I, and Figure S23A). Concordantly, FAP expression was markedly upregulated in tumour relative to normal tissue (*p* < 0.01; Figure S24A), and this increase was further confirmed in paired bladder cancer samples (*p* < 0.01; Figure S24B). Prognostic analysis further demonstrated that high FAP expression was significantly associated with worse OS, DSS, and PFI (OS: Log‐rank *p* < 0.001; DSS, Log‐rank *p* = 0.005; PFI, Log‐rank *p* = 0.043; Figure S24C‐E). Cell–cell communication analysis similarly showed that both the number and strength of interactions between FAP^+^ CAF and MPS‐MP activated malignant cells were substantially greater than those observed with tumour cells lacking MP4 program activation (Figure 3J). Ligand‐receptor interaction mapping highlighted multiple extracellular matrix and adhesion‐related signalling axes between FAP^+^ CAF and malignant cells (Figure 3K). Additionally, scDRS analysis confirmed FAP^+^ CAF and malignant cells illustrated higher risk score, suggesting that FAP^+^ CAF promoted tumour progression through matrix remodelling and reciprocal signalling (Figure S25A,B).

Extending these molecular and prognostic insights to spatial and functional contexts, we further characterized the spatial distribution of FAP^+^ CAF and tumour cells^10^ (Figure S26A), and found that FAP^+^ CAF high‐expression regions exhibited pronounced co‐localization and coordinated enrichment with malignant tumour cells (Figure 4A, and Figure S27A–C). Immunohistochemistry provided histological validation of these findings, showing maximal FAP, PDPN, POSTN and COL1A1 expression in MIBC samples (Figure 4B and Figure S28A). Flow cytometric analysis also supports a progressive accumulation of FAP^+^ CAFs with increasing malignant progression of bladder cancer (Figure S29A). Primary fibroblasts were isolated and characterized by flow cytometric sorting and immunofluorescence (Figure S29A,B), and co‐culture systems were established using NFs or FAP^+^ CAFs with bladder cancer cells. CCK‐8 assays showed that, compared with NF, FAP^+^ CAF co‐culture significantly enhanced T24 bladder cancer cell proliferation (Figure 4C), which was further confirmed by colony formation assays (Figure 4D). Three‐dimensional spheroid assays likewise demonstrated that FAP^+^ CAF co‐culture increased tumour spheroid‐forming capacity (Figure 4E). Transwell assays further showed that relative to NF, FAP^+^ CAF markedly increased tumour cell migration and invasion (Figure 4F). In parallel, T24 cells exposed to FAP^+^ CAFs exhibited significantly elevated expression of stemness‐associated markers, including SOX2, OCT4, ALDH1A1, CD133 and CD44 (Figure 4G and Table S4), indicating acquisition of a more stem‐like and aggressive phenotype. Importantly, silencing FAP in CAFs attenuated these tumour‐promoting effects, as evidenced by reduced proliferative activity, colony formation, and invasive behaviour of co‐cultured T24 cells (Figure 4H–J and Figure S30). Ultimately, co‐injection of FAP^+^ CAF with T24 cells in a mouse xenograft model accelerated tumour formation and significantly increased terminal tumour volume and weight compared with T24 + NF cells (Figure 4K). Together, these results demonstrate that FAP^+^ CAF potentiates malignant proliferation, migration and invasion of bladder cancer cells, thereby promoting disease progression.

**FIGURE 4:**
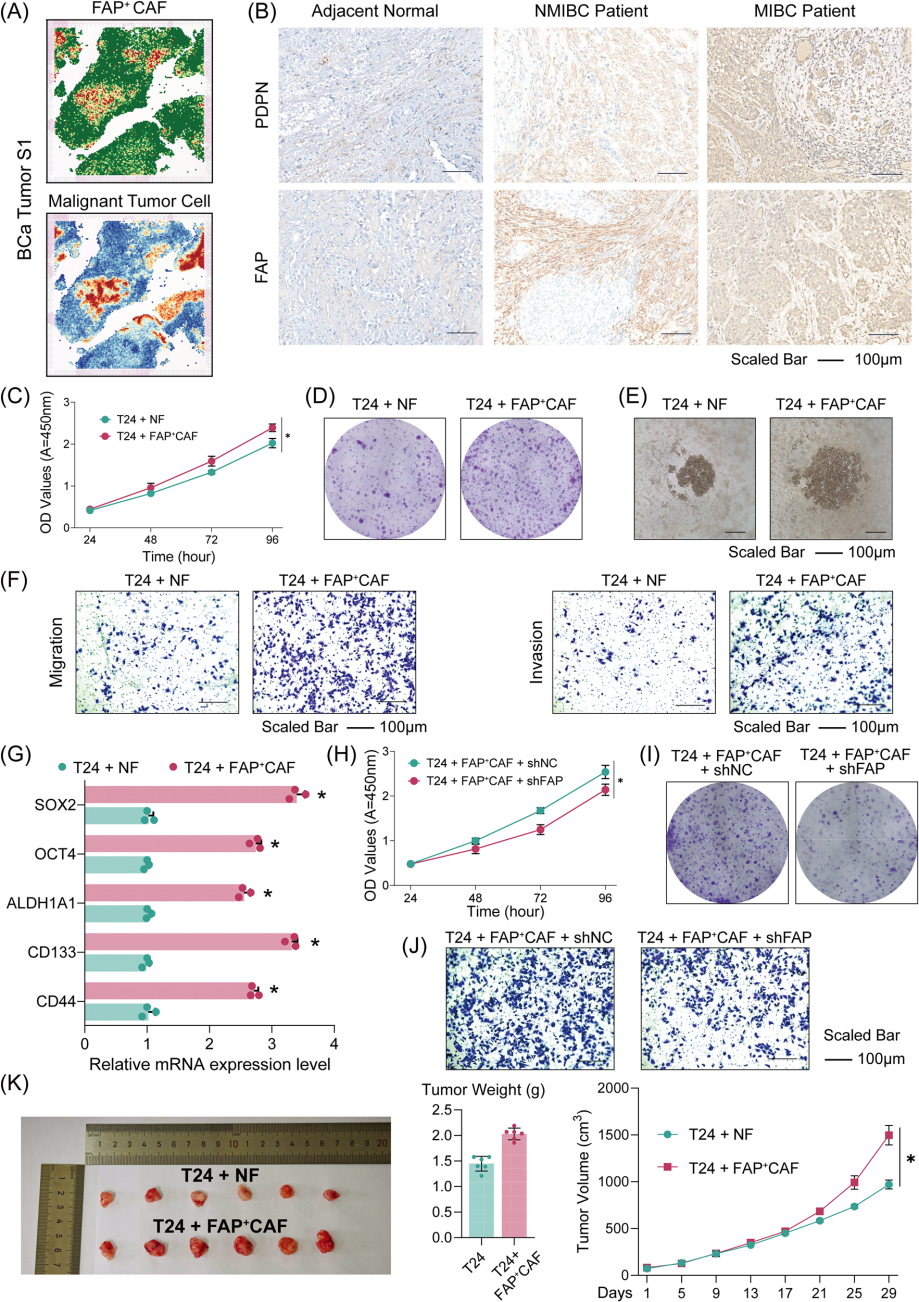
Spatial profiling and functional experiments validated the pro‐tumourigenic role of FAP^+^ CAFs in bladder cancer progression. (A) Spatial transcriptomes of bladder cancer specimens showing the distribution of FAP^+^ CAFs and malignant tumour cells in sample S1. (B) Representative immunohistochemical staining of FAP^+^ CAFs marker genes (PDPN and FAP) in adjacent normal bladder tissue and NMIBC/MIBC bladder cancer tissue. Scale bars = 100 µm. (C) CCK‐8 cell proliferation assay of T24 bladder cancer cells co‐cultured with normal fibroblasts (NF) or FAP^+^ CAFs. (D) Representative colony‐formation assay showing that T24 cells exposed to NF or FAP^+^ CAFs conditions. (E) Representative tumour sphere formation images of co‐culture assays. Scale bars = 100 µm. (F) Representative Transwell migration and invasion assays of T24 cells cultured with NF or FAP^+^ CAFs. Scale bars = 100 µm. (G) Representative mRNA expression level of T24 cells cultured with NF or FAP^+^ CAFs. (H) CCK‐8 cell proliferation assay of T24 cells cultured with FAP^+^ CAFs transfected with negative control (shNC) or FAP‐targeting shRNA (shFAP). (I) Representative images of colony formation assays of T24 cells co‐cultured with FAP‐silenced CAFs compared with control CAFs. (J) Transwell migration assays reveal that knockdown of FAP in CAFs markedly suppresses the migratory potential of T24 cells. Scale bar = 100 µm. (K) In vivo xenograft validation of the tumor‐promoting effect of FAP^+^ CAFs (*N* = 6). Data are presented as mean ± SD from at least three independent experiments. *: *p* < 0.05.

In summary, this research identified a tumour‐stroma niche driven by FAP^+^ CAFs and MPS‐MP activated malignant epithelial cells as a central driver of bladder cancer progression. This interactive axis orchestrates enhanced proliferative stemness, EMT, hypoxia responses, and aberrant intercellular signalling, culminating in aggressive invasion and poor clinical outcome. Multi‐modal evidence, spatial co‐localization, and in vitro/in vivo models establish FAP^+^ CAFs as dominant pro‐tumorigenic stromal determinants. Further validation and prospective clinical investigations will be required to validate and extend the conclusions of the present study. Collectively, these findings delineate the FAP^+^ CAF–MPS‐MP axis as both a mechanistic cornerstone of muscle‐invasive progression and a promising therapeutic vulnerability in bladder cancer (Figure 5).

**FIGURE 5:**
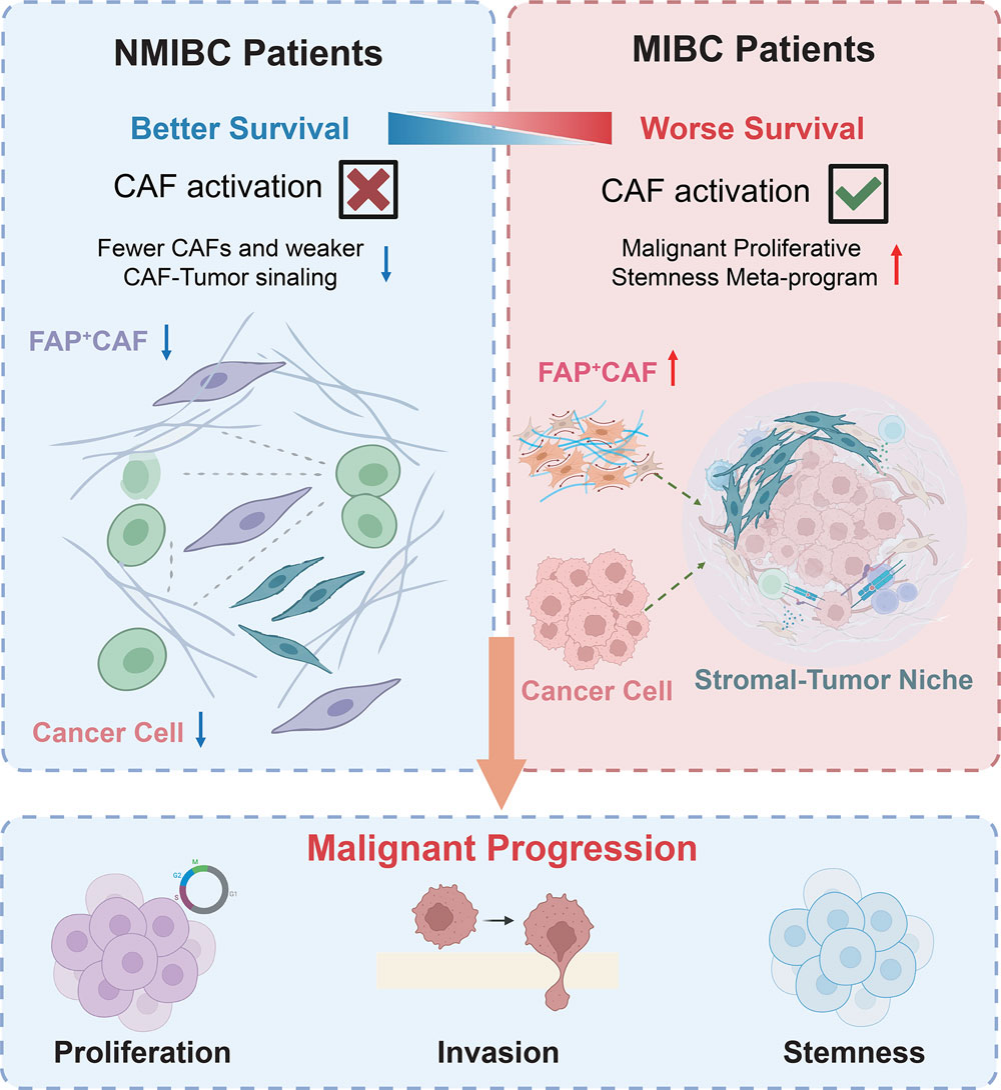
Schematic diagram of the mechanism proposed in this study.

## AUTHOR CONTRIBUTIONS

M.L. Wang and J.Y. Xin conceived of the study and carried out its design. J.J. Wu and B.X. Liu performed analysis, experiments and wrote the paper. S.L. Chen and M.L. Wang revised the paper. All authors read and approved the final version of this manuscript.

## FUNDING INFORMATION

The authors have nothing to report.

## CONFLICT OF INTEREST STATEMENT

The authors declare no conflicts of interests.

## ETHICS STATEMENT AND CONSENT TO PARTICIPATE

This study involving human participants and clinical samples was approved by Nanjing Medical University Institutional Ethics Committee. Participants signed informed consent to participate in the study. Animal experiments involved in this research were approved by the Institutional Animal Care and Use Committee (IACUC) of Nanjing Medical University.

## Supporting information



Supporting information

## Data Availability

Due to patient privacy restrictions, single‐cell RNA sequencing data from the NJBC cohort are available from the corresponding author upon reasonable request. Detailed sources of the remaining publicly available single‐cell RNA sequencing datasets from normal bladder tissues and bladder cancer samples are provided in the Methods section and Supporting Information. TCGA bladder cancer RNA‐sequencing data and the corresponding clinical information were downloaded from the TCGA database. Other data relevant to this study are available from the corresponding author upon reasonable request.
