# Supplementary material for: Stromal FAP+ cancer associated fibroblasts orchestrate a pro‐tumorigenic niche with malignant proliferative stemness and cancer progression
Source: Clin Transl Med. 2026 May 13;16(5):e70688. doi: 10.1002/ctm2.70688 (PMC13168937; doi:10.1002/ctm2.70688)
Supplement: Supplementary file 1 — Supporting information [file CTM2-16-e70688-s001.pdf]

Supplementary Materials and Methods

Supplementary Methods

Supplementary Figures

Figure S1. Quality-control of the single-cell RNA-sequencing dataset.

Figure S2. Cell distribution of the integrated bladder cancer scRNA atlas.

Figure S3. Overview of bladder cancer meta-GWAS signals and the scDRS-based analytical framework for single-cell trait association.

Figure S4. Integrative identification of malignant epithelial cells.

Figure S5. Consensus non-negative matrix factorization identifies recurrent epithelial metaprograms in bladder cancer scRNA atlas.

Figure S6. Enrichment analysis results of “Hallmark” and “CancerSEA” gene programs for each MP program.

Figure S7. cNMF-defined epithelial metaprograms exhibit distinct activation patterns in non-muscle-invasive and muscle-invasive bladder cancer.

Figure S8. “DUBstepR”-based re-clustering reveals distinct epithelial cell landscapes between NMIBC and MIBC.

Figure S9. Comparative expression analysis of essential marker genes of the MPS-MP across NMIBC/MIBC, NMIBC/MIBC specific, and MPS-MP inactivated/activated cell subpopulations.

Figure S10. Transcription factor enrichment analysis using “CollecTRI” database, depicting ATF3 as a key regulator among MPS-MP activated cells.

Figure S11. Functional enrichment and pathway annotation of MPS-MP activated cells.

Figure S12. Cell-cell communication analysis between MPS-MP activated/inactivated cells and other stromal and immune cells.

Figure S13. “scSurv” framework identifies survival-associated cellular programs to clinical survival risk.

Figure S14. Single-cell mapping of TCGA-derived survival-associated signatures across major cell populations in the tumor microenvironment.

Figure S15. Scissor analysis identifies cell populations associated with multiple survival endpoints in bladder cancer.

Figure S16. Elevated CAF-related stromal signatures are consistently associated with

unfavorable survival in bladder cancer across independent cohorts.

Figure S17. Comprehensive intercellular communication analysis of bladder cancer TME.

Figure S18. Single-cell transcriptomic landscape of fibroblast populations in bladder cancer.

Figure S19. Disease stage dependent remodeling of CAF subpopulations in bladder cancer.

Figure S20. Prognostic-related signatures are differentially enriched across CAF subpopulations.

Figure S21. Scissor analysis links distinct CAF states to survival outcomes in bladder cancer.

Figure S22. Functional characterization of CAF subpopulations based on “CancerSEA” and “Hallmark” gene-set enrichment analyses.

Figure S23. Kaplan-Meier survival analyses in the merged GEO bladder cancer cohort stratified by FAP^{+} CAFs enrichment level.

Figure S24. Elevated FAP expression in tumors is associated with poor clinical outcomes.

Figure S25. Violin plots showing the distribution of bladder cancer scDRS score across MPS-MP activated/inactivated and fibroblast cell subpopulations.

Figure S26. Quality assessment of spatial transcriptomic data in bladder cancer tumor specimens.

Figure S27. Spatial distribution and correlation analysis of FAP^{+} CAFs and malignant epithelial tumor cells.

Figure S28. Immunohistochemical staining of POSTN, and COL1A1 in adjacent normal bladder tissue and NMIBC/MIBC bladder cancer tissue. Scale bars = 100 μm.

Figure S29. Flow-cytometric isolation and phenotypic validation of FAP^{+} CAFs.

Figure S30. Validation of FAP shRNA knockdown efficiency in primary FAP^{+} CAFs.

Supplementary Tables

Table S1. Clinicopathological characteristics for Nanjing bladder cancer cohort.

Table S2. Public datasets used in this research.

Table S3. Differently expressed genes in MPS-MP activated cells compared with MPS-MP inactivated cells.

Table S4. Oligonucleotide sequences used in this research.

Supplementary Methods

Tissue dissociation and single-cell sequencing

In the Nanjing bladder cancer cohort, we collected paired adjacent normal bladder tissues and tumor tissues from treatment-naïve patients undergoing radical cystectomy. Samples were obtained from Jiangsu Provincial People's Hospital and Jiangsu Provincial Hospital of Traditional Chinese Medicine. Adjacent normal tissues were defined as histologically normal bladder mucosa located > 5 cm from the tumor margin. All diagnoses were independently confirmed by at least two senior pathologists. Written informed consent was obtained from all patients prior to tissue collection. The study involving human participants and clinical specimens was approved by Nanjing Medical University and the participating hospitals and was conducted in accordance with the principles of the Declaration of Helsinki. Detailed clinicopathological information of the patients is provided in **Tab. S1**.

Freshly resected tissues were immediately placed in sterile dishes containing 10 mL ice-cold 1× Dulbecco's phosphate-buffered saline (DPBS) to remove residual preservation solution. Tissues were then mechanically minced on ice. Enzymatic dissociation was performed in a digestion buffer consisting of 0.25% trypsin and 10 μg/mL DNase I in PBS supplemented with 5% fetal bovine serum (FBS). Digestion was carried out at 37 °C with 50 rpm for approximately 40 min. To maximize cell yield and viability, released cells were collected every 20 min, and fresh digestion buffer was added to the remaining tissue fragments. The resulting cell suspensions were filtered through a 40-μm cell strainer to remove debris, followed by red blood cell lysis using 1× RBC Lysis Solution. Cells were washed twice in 1× DPBS containing 2% FBS, stained with 0.4% Trypan Blue, and assessed for viability and concentration using a Countess II Automated Cell Counter to ensure they met the quality thresholds for downstream single-cell library construction.

Single-cell barcoding was achieved on the 10× Genomics platform by co-encapsulating single cells with gel beads carrying unique molecular identifiers (UMIs) and cell barcodes in Gel Beads-in-Emulsion (GEMs) under near-saturating conditions. Following cell lysis, polyadenylated mRNA hybridized to oligonucleotides on the bead surface. All beads were recovered into a single tube for reverse transcription, during which cDNA

molecules were tagged at the 5′ end with both UMI and cell barcode, enabling single-cell resolution tracking. Subsequent steps included second-strand synthesis, adapter ligation, and universal amplification. Sequencing libraries were constructed from fragmented whole-transcriptome amplification products, with enrichment of 3′ fragments carrying cell barcodes and UMIs. Library preparation strictly followed the manufacturer's CG000206 Rev D protocol. Final libraries were evaluated for fragment size distribution using a High Sensitivity DNA Chip on a Bioanalyzer 2100 system and quantified with the Qubit High Sensitivity DNA Assay. Sequencing was performed on an Illumina NovaSeq 6000 platform in paired-end mode.

Single-cell RNA sequencing analysis

(1) Gene expression matrix generation and quality control

We integrated scRNA-seq data from normal bladder, adjacent normal, and bladder tumor tissues, including the NJBC cohort and publicly available datasets (CNP0000460, HRA003620, PRJNA662018, GSE135337, GSE217956, GSE211388, PRJNA609439, GSE222315, GSE130001, GSE192575, Salome's cohort, HCA cohort, GSE129845, and GSE159929). Detail information of datasets used in this research is listed in **Tab. S2**. Only treatment-naive samples collected prior to any therapeutic intervention were included. For bladder cancer tissues, only samples with available clinical annotation regarding muscle invasiveness were retained. Raw sequencing data were processed using CellRanger (v8.0.1) with default parameters. Low-quality cells were excluded if they had fewer than 200 detected genes, fewer than 500 UMI counts, > 10% mitochondrial gene content, or > 10% hemoglobin-related gene expression. Cells with total transcript counts or detected gene numbers exceeding 5× the median absolute deviation (MAD) were also removed. Raw count matrices were subsequently processed and merged using the "Scanpy" Python package (v1.12.1). Potential doublets were identified and removed using the deep-learning-based SOLO algorithm. After count normalization and log-transformation for each sample, the top 3,000 highly variable genes were selected. Data integration was performed using the "scVI" algorithm to correct for potential batch effects, with dataset source, patient ID, and sequencing platform modeled as covariates.

(2) Cell clustering and subpopulation annotation

Dimensionality reduction was performed by projecting scVI latent embeddings onto

two-dimensional space using Uniform Manifold Approximation and Projection (UMAP). Cell clusters were identified with the Leiden algorithm. For malignant epithelial cells, we applied the "DUBStepR" algorithm to identify informative gene sets for epithelial sub clustering. Tumor cells were then re-clustered using these feature genes.

(3) Cell distribution preference analysis

To systematically evaluate distributional biases of cell subpopulations across phenotypic groups or tissue origins, we quantified cell numbers and relative proportions per sample. Enrichment trends and preference strength were assessed using the Ro/e index (observed-to-expected ratio) and odds ratio (OR). Ro/e > 1 or OR > 1 indicated relative enrichment in a given group, while Ro/e < 1 or OR < 1 indicated depletion. Density plots were used to visualize overall proportional differences. Statistical significance of proportional differences across multiple groups was evaluated using the "scProportion" R package with sensitivity analyses.

(4) Identification of malignant epithelial cells

Malignant cells were first identified using Cancer-Finder, a domain-generalization deep-learning algorithm designed to improve accuracy and generalizability of malignancy annotation across pan-cancer single-cell datasets. Large-scale copy number variation (CNV) inference was performed on epithelial cells using the "InferCNV" R package with a hidden Markov model (HMM), applying parameters "denoise" and cutoff = 0.1. CNV scores were calculated as $\sum[(CNV_{i-1})^2]$, where CNV_i represents the inferred copy number level of chromosomal region i. Epithelial cells from patient-matched adjacent normal tissues or immune cells from the same patient served as reference. K-means hierarchical clustering was applied to CNV signals and scores to distinguish malignant populations. CNV scores derived from the "InferCNV" output matrix quantified genome-wide deviation; higher scores indicated greater CNV burden and stronger malignant potential. Cells classified as malignant by both "Cancer-Finder" and "InferCNV" clustering were defined as malignant epithelial cells.

(5) Functional enrichment and pathway annotation

Pathway activity scores for individual cells were computed using the "AUCell" R package by calculating the area under the curve (AUC) of the ranked expression of target gene sets. Cells were subsequently stratified according to "AUCell"-derived AUC

thresholds to determine meta-program activation status, whereby cells with AUC values exceeding the threshold were classified as active, and those below the threshold as inactive. Gene sets were sourced from “Hallmark” collections and “CancerSEA” functional state signatures.

(6) Tumor expression meta-program (MP) identification

To dissect intra-tumoral transcriptional heterogeneity in malignant bladder epithelial cells and extract interpretable expression meta-programs, we employed a consensus non-negative matrix factorization (cNMF) strategy. Malignant cells were decomposed on a per-sample basis; only samples containing > 200 malignant cells were included to ensure decomposition stability. For each qualifying sample, 3,000 highly variable genes were selected after preprocessing and scaling, with mitochondrial genes excluded to reduce stress or technical noise driven artifacts. Consensus NMF was performed across factorization ranks 2-10 to assess program stability and reproducibility. The top 50 genes with highest factor weights per program served as signature vectors. Robust, cross-sample reproducible programs were selected by first retaining factors with standard deviation > 0.2 across cells. Jaccard similarity was then calculated between program gene-weight or exposure vectors to identify biologically coherent program clusters. Based on the “AUCell” scoring scheme, cells with an MPS-MP enrichment AUC value > 0.25 were considered to exhibit MPS-MP activation, whereas the remaining cells were classified as non-activated.

(7) Cell–cell communication analysis

Potential intercellular communication within the bladder cancer immune microenvironment was inferred using the “CellChat” algorithm. “CellChat” leverages a curated ligand–receptor database to compute signaling pathways based on expressed ligands and receptors. Differential interaction strengths across groups were compared to identify key communication pathways and their potential regulatory roles in the tumor immune microenvironment.

(8) Single-cell Disease Relevance Score (scDRS) analysis

To map complex-disease genetic risk onto single-cell transcriptomes, we applied the “scDRS” framework, integrating scRNA-seq data with GWAS summary statistics. Gene-level associations were first computed using MAGMA with a SNP-wide mean model,

accounting for linkage disequilibrium (LD) from the 1000 Genomes Project Phase 3 reference panel. The top 1,000 genes with the strongest Z-scores were selected to construct disease-associated gene sets. These sets were matched to each cell's expression profile against a background gene set to generate normalized scDRS scores. Higher scores indicate greater transcriptional alignment with disease genetic risk. Additionally, gsMap was used with default parameters to project disease-associated GWAS signals onto spatial transcriptomic data.

(9) Scissor algorithm analysis

To identify cell subpopulations associated with clinical outcomes in bladder cancer, we applied the Scissor algorithm to integrate scRNA-seq data with TCGA-BLCA survival data. Reference phenotypes included overall survival (OS), disease-specific survival (DSS), and progression-free interval (PFI) from patients with complete follow-up. Proportions of Scissor^{+} versus Scissor^{-} cells were quantified and compared across cell types to evaluate their relative contributions to prognosis-associated transcriptional programs.

Bulk-RNA transcriptome analysis

We curated and integrated bladder cancer bulk RNA-seq datasets with complete survival information (GSE13507, GSE31684, GSE32894, GSE48075, GSE48276, GSE69795, and GSE70691). Batch effects were removed using "ComBat", with principal component analysis confirming effective correction. Bulk RNA-seq data were deconvoluted using "BayesPrism", with the above-described bladder cancer single-cell atlas (tumor samples) serving as the single-cell reference to estimate cellular composition. For cross-validation, multiple marker-gene-based deconvolution and scoring algorithms implemented in the "IOBR" R package (EPIC, MCPcounter, and ESTIMATE) were applied to assess CAF abundance and tumor microenvironment features. Associations between algorithm-derived CAF metrics and patient survival outcomes were systematically evaluated to delineate the prognostic relevance of CAFs in bladder cancer.

Spatial transcriptome analysis

To reveal the spatial distribution of cellular subpopulations in bladder cancer microenvironment, we performed spatial transcriptome analysis. Spatial transcriptomic

data underwent unsupervised clustering using the "STAGATE" algorithm, followed by targeted denoising to reconstruct high-fidelity gene expression profiles. Cell-type deconvolution was subsequently performed with the "Tangram" algorithm, integrating matched single-cell RNA-sequencing reference profiles to infer the spatial distribution and relative abundance of distinct cellular compartments. Correlation of cell-type proportions within individual spots was quantitatively assessed using Spearman's correlation coefficient.

Subcutaneous tumorigenesis assay in nude mice

4-6 weeks old Female BALB/c-Nude mice were housed under specific pathogen-free conditions. After 1 week of acclimatization, mice were randomized into two groups: T24 + NF, T24 + FAP^+ CAF. Defined numbers of T24 bladder cancer cells were mixed with normal fibroblasts (NF) or cancer-associated fibroblasts (CAF) at predetermined ratios, resuspended in serum-free medium, and combined 1:1 with Matrigel. The mixture was injected subcutaneously into the axillary region. Tumor growth was monitored daily, and tumor dimensions (length L, width W) were measured with digital calipers every 3 days. Tumor volume was calculated as $V = (L \times W^2)/2$. Tumor formation time, growth kinetics, and body weight were recorded. Experiments were terminated upon reaching ethical endpoints. Mice were euthanized under anesthesia, and tumors were excised, weighed, and photographed.

Isolation and fluorescence-activated cell sorting of primary CAFs

Fresh bladder tumor tissues and matched adjacent normal bladder tissues were collected, rinsed in ice-cold antibiotic-containing PBS, and minced into about 1 mm^3 fragments. Tissues were digested with type I collagenase at 37 °C for 1.5 hours with gentle pipetting every 15 min. Digestion was terminated with serum-containing medium when fragments loosened. Single-cell suspensions were obtained by 70 μm filtration, centrifuged, and resuspended in FACS buffer. After Fc receptor blocking and live/dead staining, cells were incubated with fluorochrome-conjugated antibodies at 4 °C in the dark. Firstly, we employed a tumor cell CD45 negative selection kit to exclude CD45 positive cells. Gating strategy: FSC/SSC for singlets, exclusion of dead cells, followed by lineage-negative selection ($CD45^-CD31^-EPCAM^-$) to enrich fibroblasts. Within the tumor-derived $CD45^-CD31^-EPCAM^-$ population, FAP^+PDPN^+ CAFs were sorted. Sorted

cells were collected into tubes pre-filled with complete medium, centrifuged, and plated. Culture medium was replaced after 24 h to remove residual antibodies and debris. Purity of sorted NF, and FAP^{+} CAF populations was validated by immunofluorescence for COL1A1, Vimentin, PDPN, and FAP.

Cell culture and transfection

The bladder urothelial carcinoma cell line T24 was purchased from the Cell Bank of the Chinese Academy of Sciences and maintained in DMEM supplemented with 10% FBS and 1% penicillin/streptomycin. All cell lines and primary cells were authenticated by STR profiling and routinely tested negative for mycoplasma. Primary cells were used within five passages.

Cell immunofluorescence assay

Cells were washed twice with PBS, fixed with 4% paraformaldehyde for 15 min at room temperature, and permeabilized with 0.1% Triton X-100 for 10 min. After blocking with 5% bovine serum albumin for 30 min, cells were incubated with primary antibodies overnight at 4 °C, followed by fluorescently labeled secondary antibodies. Nuclei were counterstained with DAPI for 10 min. Samples were mounted with anti-fade medium and imaged under a fluorescence microscope.

Tumor colony formation and 3D sphere formation assays

For sphere formation, pretreated bladder cancer cells were cultured in ultra-low attachment plates in serum-free basal medium supplemented with 20 μg B27, 20 ng/mL EGF, and 5 μg/mL insulin. Spheres were imaged and quantified by volume.

For colony formation, 1000 pretreated T24 cells were seeded in six-well plates and cultured for 10 days. Colonies were fixed with 4% paraformaldehyde and stained with 0.1% crystal violet.

Immunohistochemistry (IHC) experiment

Bladder tissues were fixed in 4% paraformaldehyde at 4 °C immediately after resection. Samples underwent dehydration, clearing, and paraffin embedding. Sections were cut, mounted on pre-treated slides, and dried at 37 °C for 1 h. Deparaffinization was performed in xylene, followed by rehydration through a graded ethanol series and rinsing in deionized water. Antigen retrieval was conducted in EDTA buffer at 95-100 °C for 15 min. After cooling and PBS washes, sections were blocked with 5% BSA for 30 min.

Primary antibodies were applied overnight at 4 °C. Following PBS washes, HRP-conjugated secondary antibodies were incubated for 1 h at room temperature. Signals were developed with 3,3′-diaminobenzidine (DAB) for 2-5 min until brown coloration appeared, followed by counterstaining with hematoxylin. Sections were dehydrated, cleared, and mounted with neutral resin for microscopic evaluation of target protein expression and intensity.

RNA extraction and reverse transcription quantitative real-time PCR (RT-qPCR) analysis

Total RNA was extracted using TRIzol reagent according to the manufacturer's instructions. Total RNA was reverse-transcribed into cDNA using HiScript III All-in-one RT SuperMix (Vazyme, China). Quantitative real-time PCR was performed using the SYBR Green detection method with SYBR qPCR Master Mix on the LightCycler 480 Real-Time PCR System (Roche, USA). All samples were run in at least triplicate technical replicates, with no-template controls included in every run to rule out contamination. Relative gene expression levels were normalized to the housekeeping gene β-actin and calculated using the $2^{-\Delta\Delta Ct}$ method. The primer sequences used in this study are listed in **Tab. S4**.

Statistical analysis

Statistical analyses were performed using R (v4.5.2) and Python (v3.10). Data are presented as mean ± standard deviation. Normally distributed continuous variables were compared using two-tailed Student's t-test; non-normally distributed variables were analyzed with the Wilcoxon rank-sum test. Kaplan–Meier survival curves and log-rank tests were used to evaluate overall survival, disease-specific survival, and progression-free interval. All experiments were performed in at least three independent replicates. A two-sided $P < 0.05$ was considered statistically significant.

Supplementary Figures

Figure S1. Quality-control of the single-cell RNA-sequencing dataset.

(A) Global distributions of single-cell quality-control metrics across all cells.

(B–D) Sample-wise distributions of nCount_RNA (B), nFeature_RNA (C), and mitochondrial gene % (D) across individual specimens.

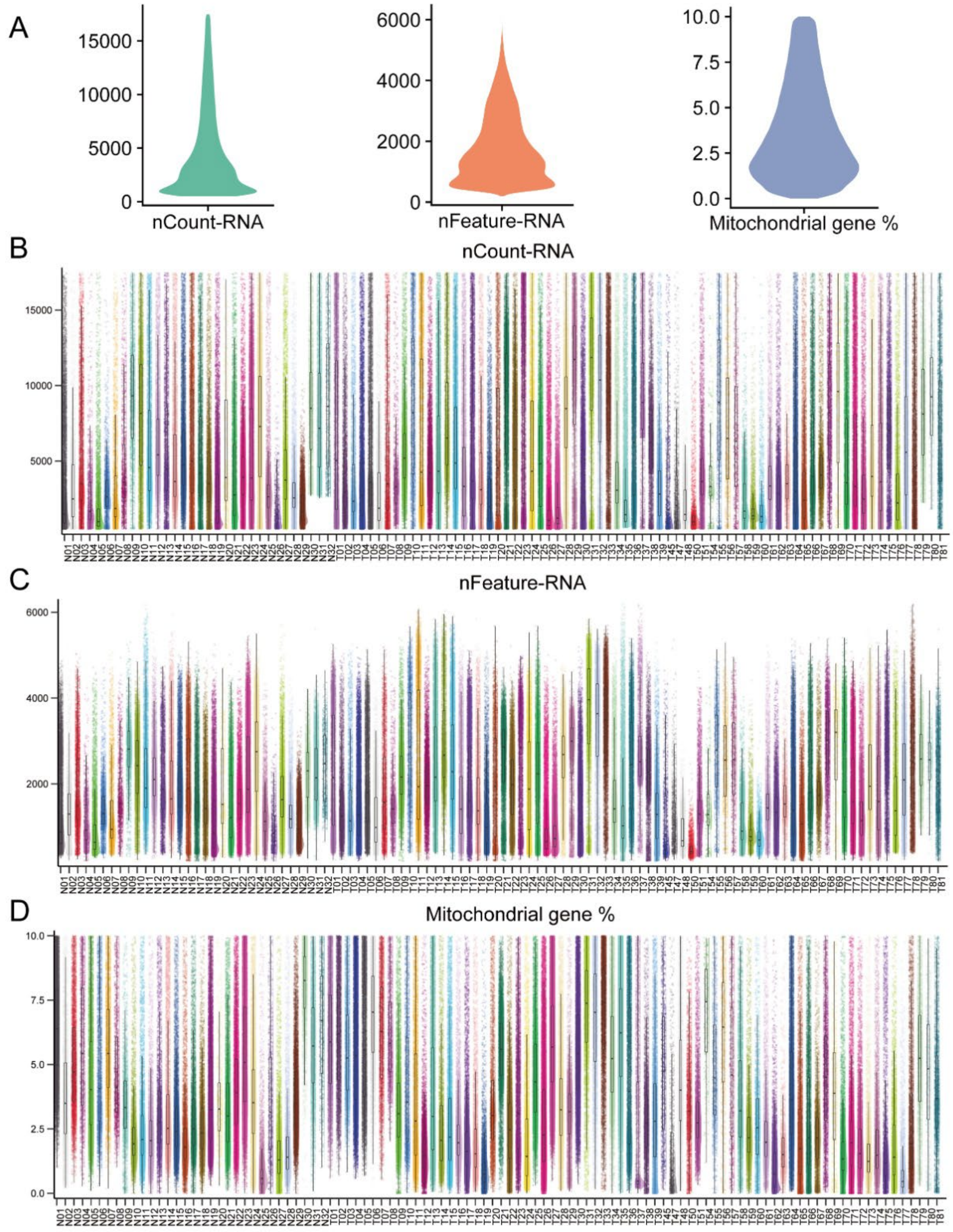

Figure S2. Cell distribution of the integrated bladder cancer scRNA atlas.

(A-C) Uniform manifold approximation and projection (UMAP) of 633,702 single cells derived from normal bladder tissues, non-muscle-invasive bladder cancer (NMIBC), and muscle-invasive bladder cancer (MIBC). Each dot represents one cell. Cells are colored according to sample category (A), library platform (B), and data cohort (C).

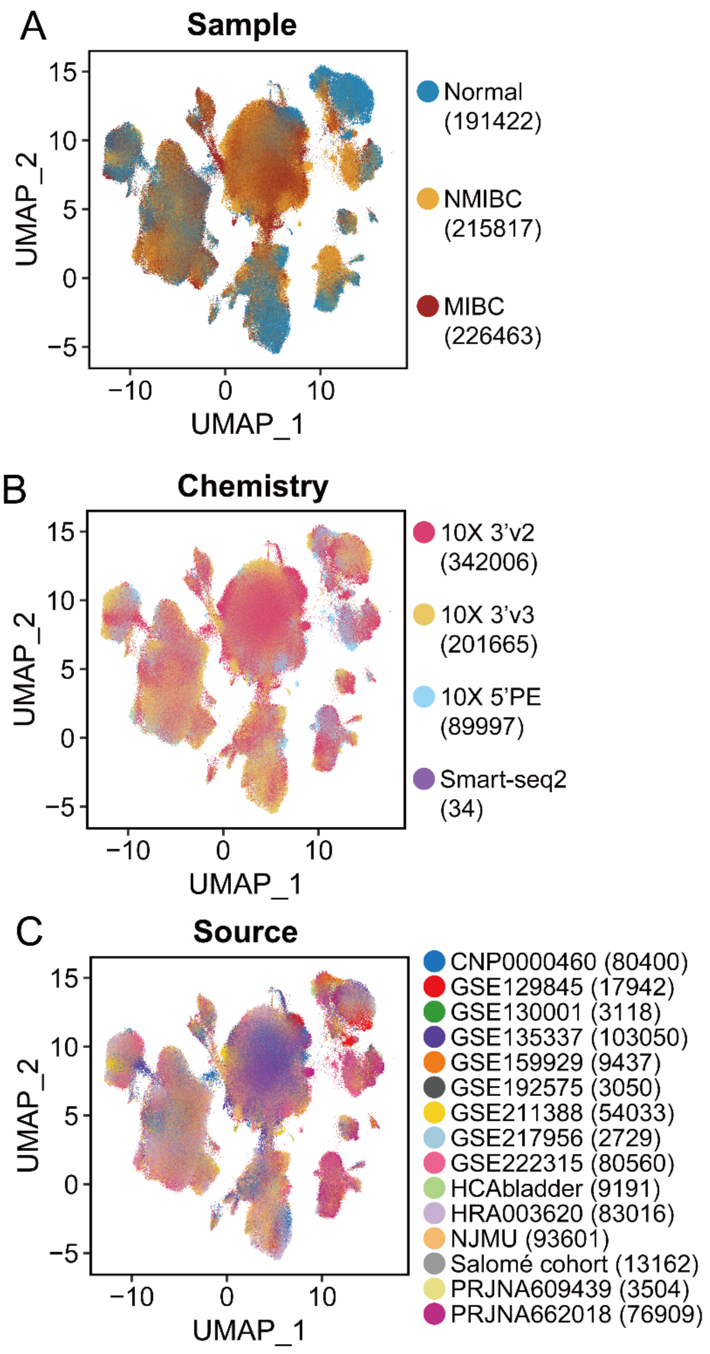

Figure S3. Overview of bladder cancer meta-GWAS signals and the scDRS-based analytical framework for single-cell trait association.

(A) The quantile–quantile (Q–Q) plot and Manhattan plot of the bladder cancer meta-genome-wide association study.

(B) Schematic illustration of the integrative analytical pipeline linking bladder cancer GWAS findings to single-cell transcriptomic data.

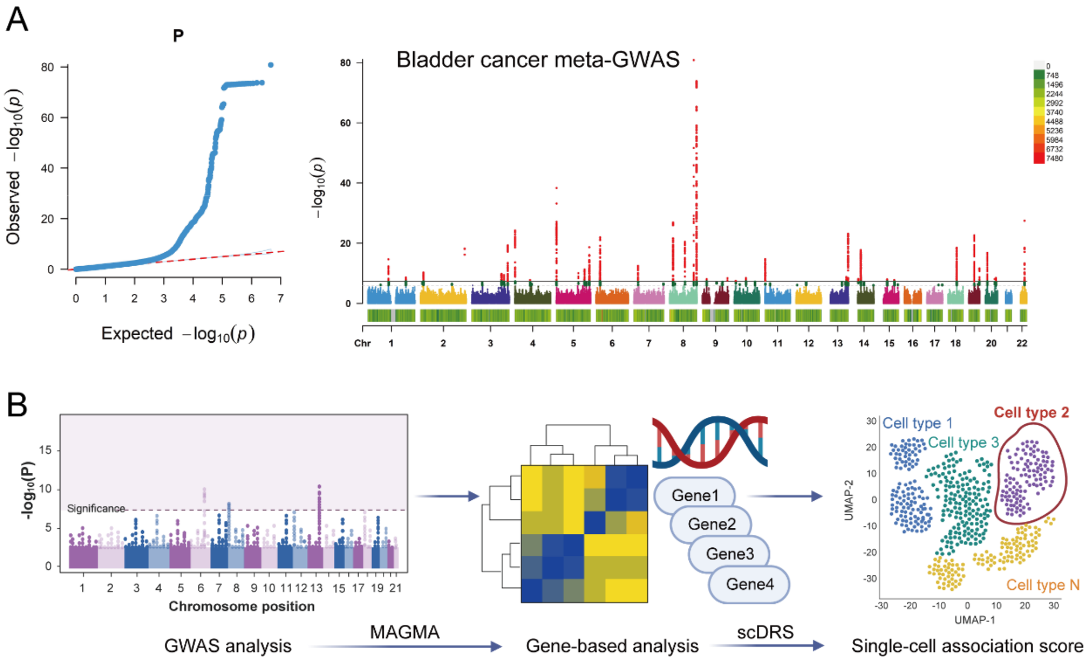

Figure S4. Integrative identification of malignant epithelial cells.

(A) Heatmap showing large-scale chromosomal copy-number variation profiles inferred by inferCNV across epithelial-cell clusters.

(B) Violin plots of inferCNV-derived CNV scores across the seven K-means clusters.

(C) UMAP visualization of epithelial cells classified by inferCNV results.

(D) UMAP projection of the final malignant-cell annotation obtained by integrating inferCNV with Cancer-Finder classification.

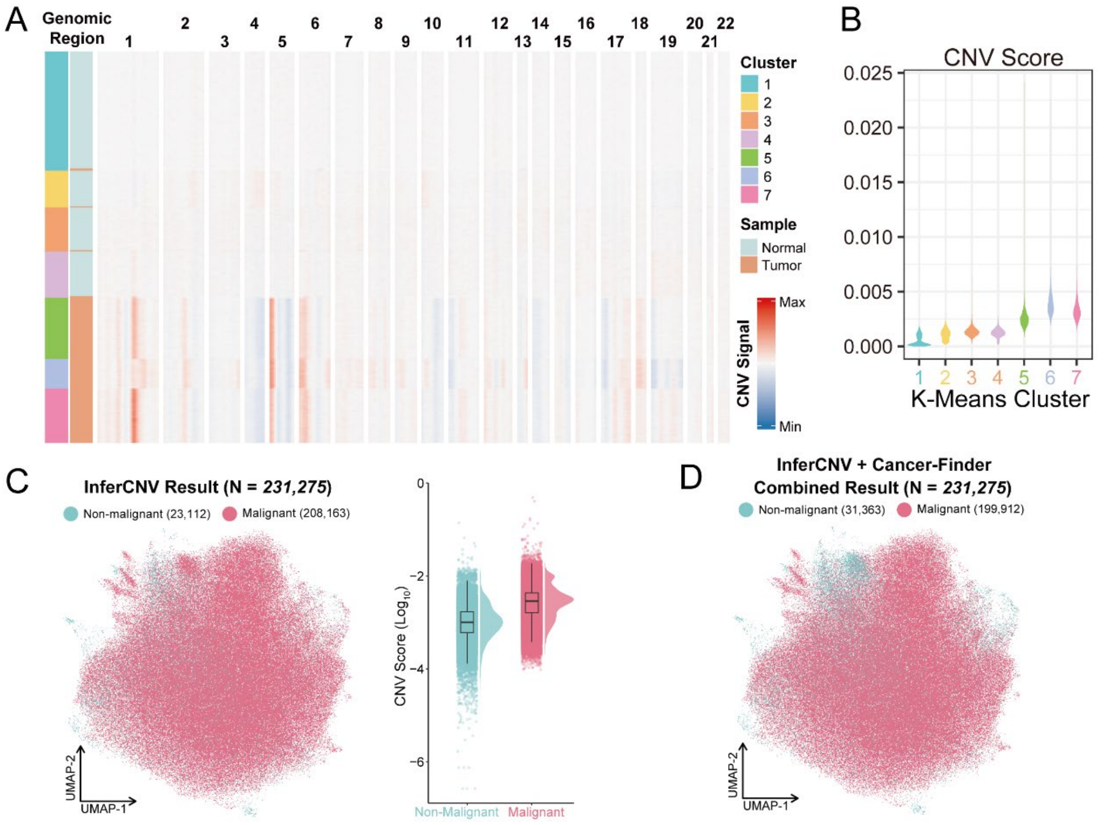

Figure S5. Consensus non-negative matrix factorization identifies recurrent epithelial metaprograms in bladder cancer scRNA atlas.

(A) UMAP feature plots showing the distribution of scaled activity scores for 11 epithelial metaprograms identified by cNMF analysis.

(B) Heatmap of representative genes defining each metaprogram across individual patients.

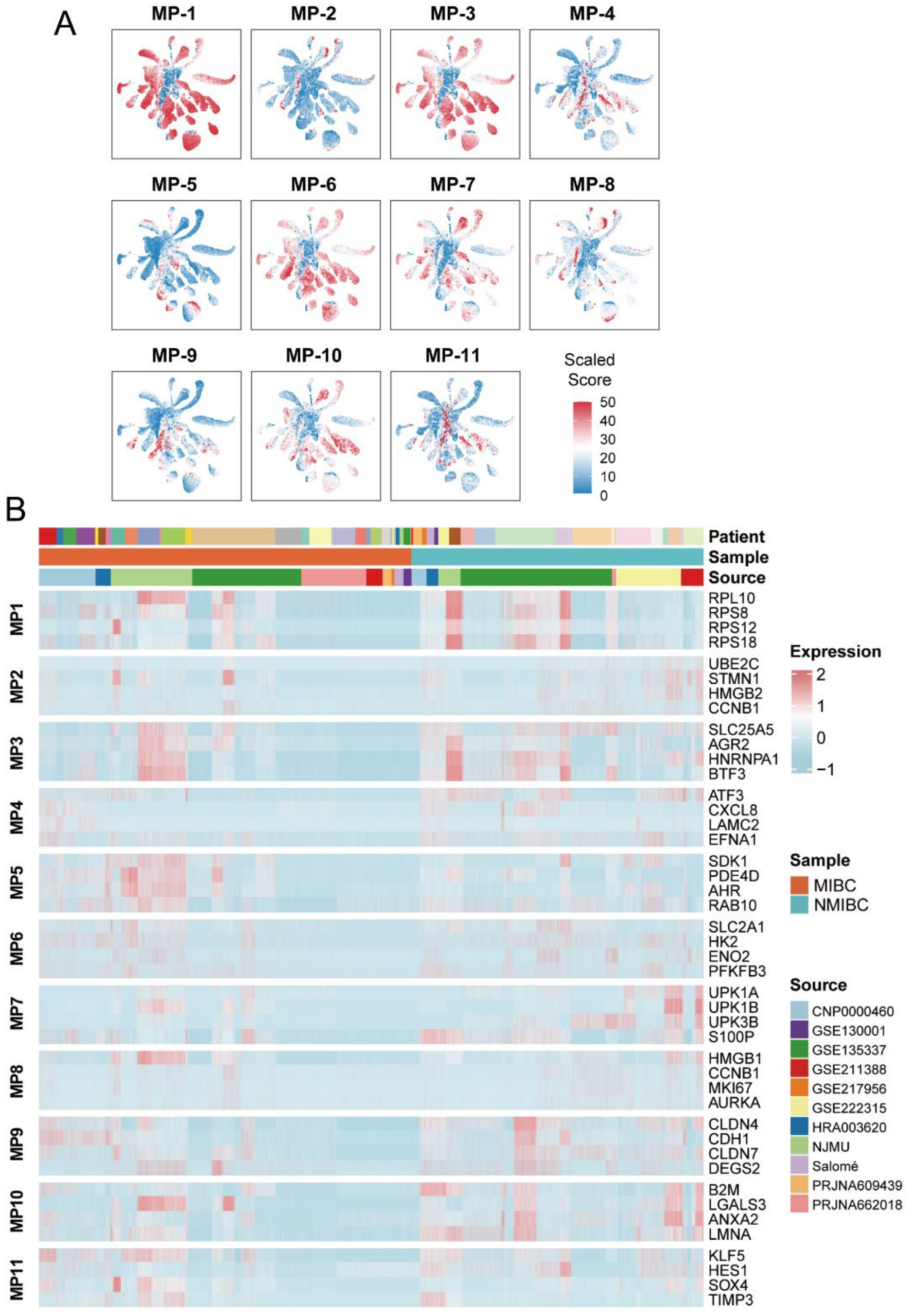

Figure S6. Enrichment analysis results of "Hallmark" and "CancerSEA" gene programs for each MP program.

(A) Functional enrichment analysis of MP 1-6.

(B) Functional enrichment analysis of MP 7-11.

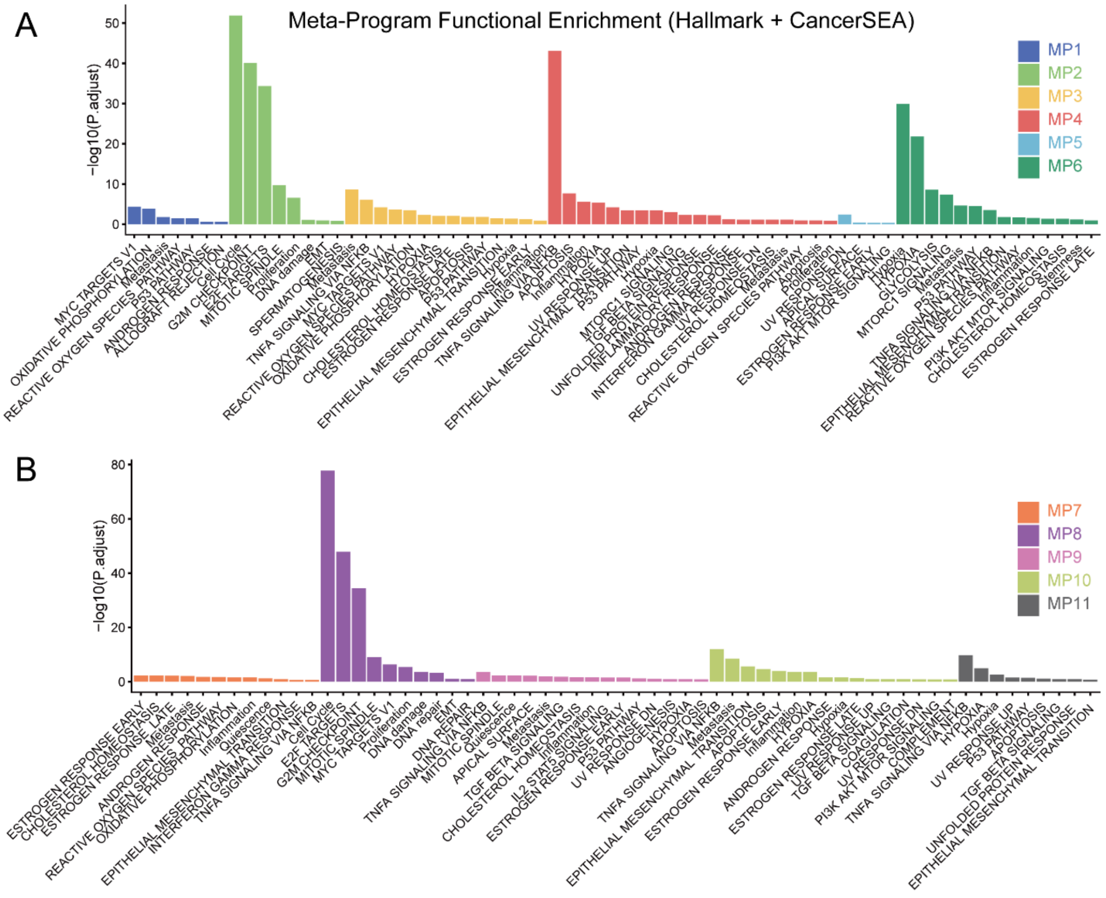

Figure S7. cNMF-defined epithelial metaprograms exhibit distinct activation patterns in non-muscle-invasive and muscle-invasive bladder cancer.

(A) Stacked bar plots showing the proportions of NMIBC- and MIBC-derived epithelial cells within the MP inactive and MP active fractions for each cNMF-derived metaprogram.

(B) UMAP plot showing the distribution of epithelial cells classified as MP active (On) or MP inactive (Off) in NMIBC and MIBC samples.

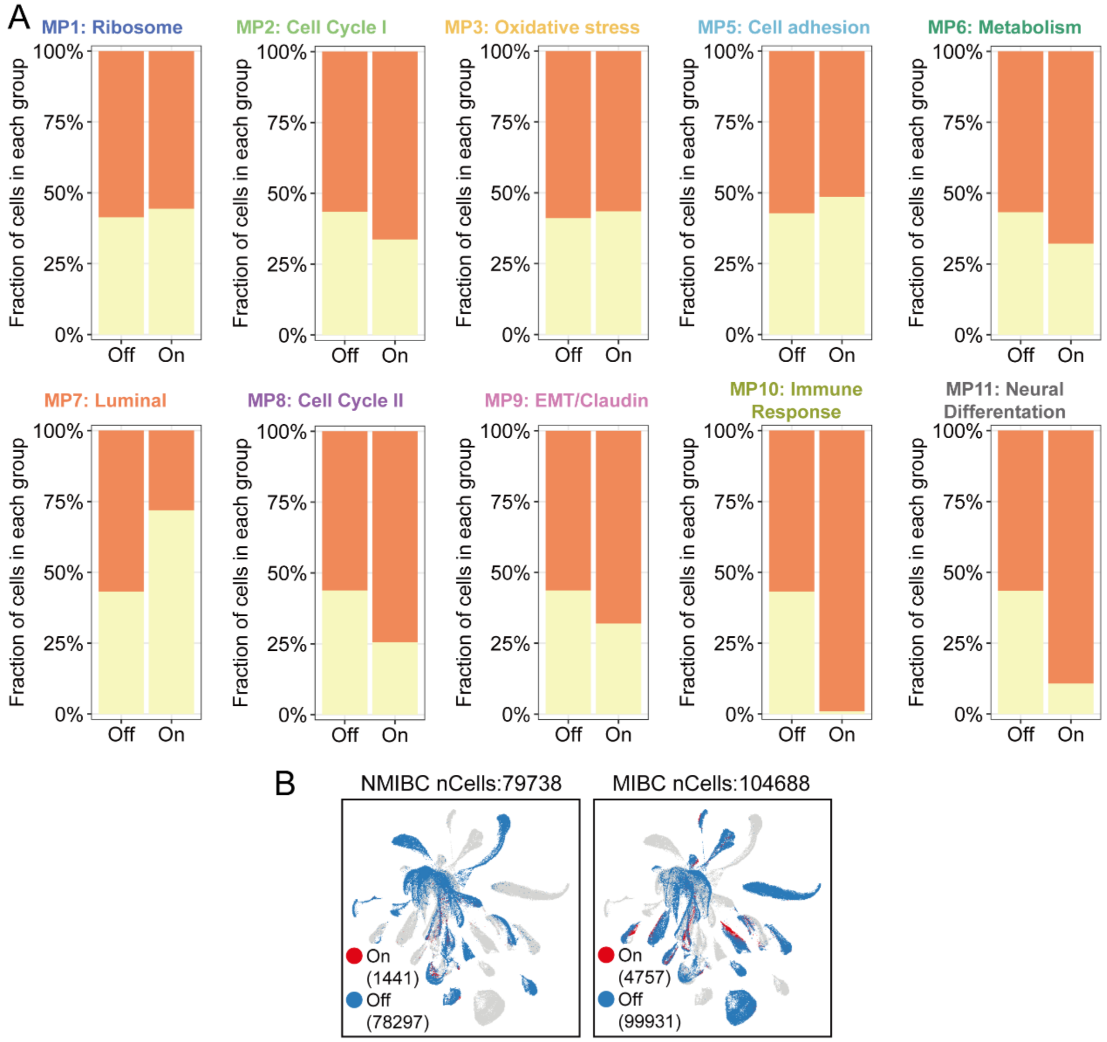

Figure S8. "DUBstepR"-based re-clustering reveals distinct epithelial cell landscapes between NMIBC and MIBC.

(A) UMAP visualization of all epithelial cells after "DUBstepR" re-clustering.

(B) Cluster-wise distribution of NMIBC- and MIBC-derived epithelial cells across the re-clustered populations.

(C) UMAP visualization of the epithelial cell compartment.

(D) Relative proportions of common, NMIBC-specific, and MIBC-specific epithelial populations in NMIBC and MIBC samples.

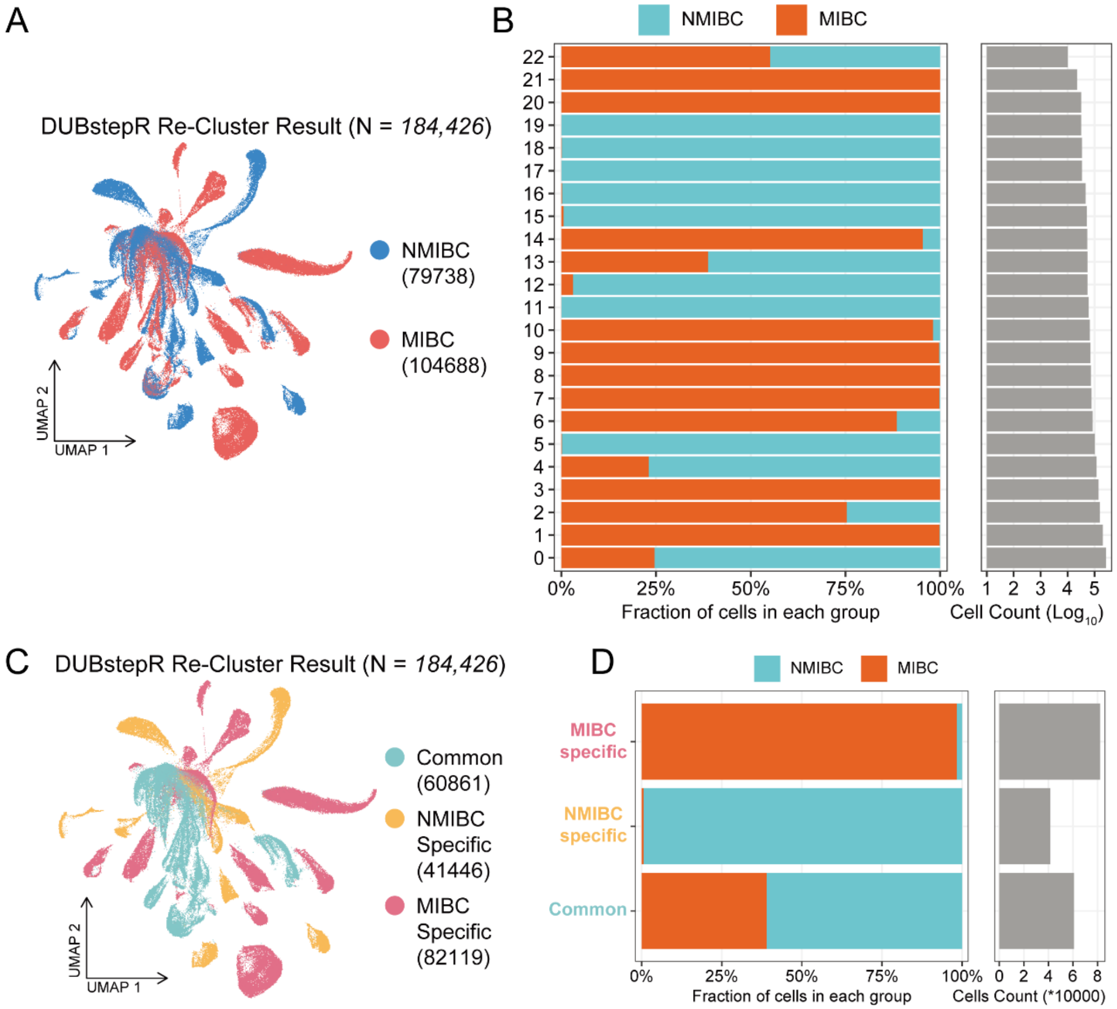

Figure S9. Comparative expression analysis of essential marker genes of the MPS-MP across NMIBC/MIBC, NMIBC/MIBC specific, and MPS-MP inactivated/activated cell subpopulations.

(A) Comparative expression analysis of MPS-MP marker genes in NMIBC/MIBC samples.

(B) Comparative expression analysis of MPS-MP marker genes in NMIBC/MIBC specific cell subpopulations.

(C) Comparative expression analysis of MPS-MP marker genes in MPS-MP inactivated/activated cell subpopulations.

****: $P < 0.0001$

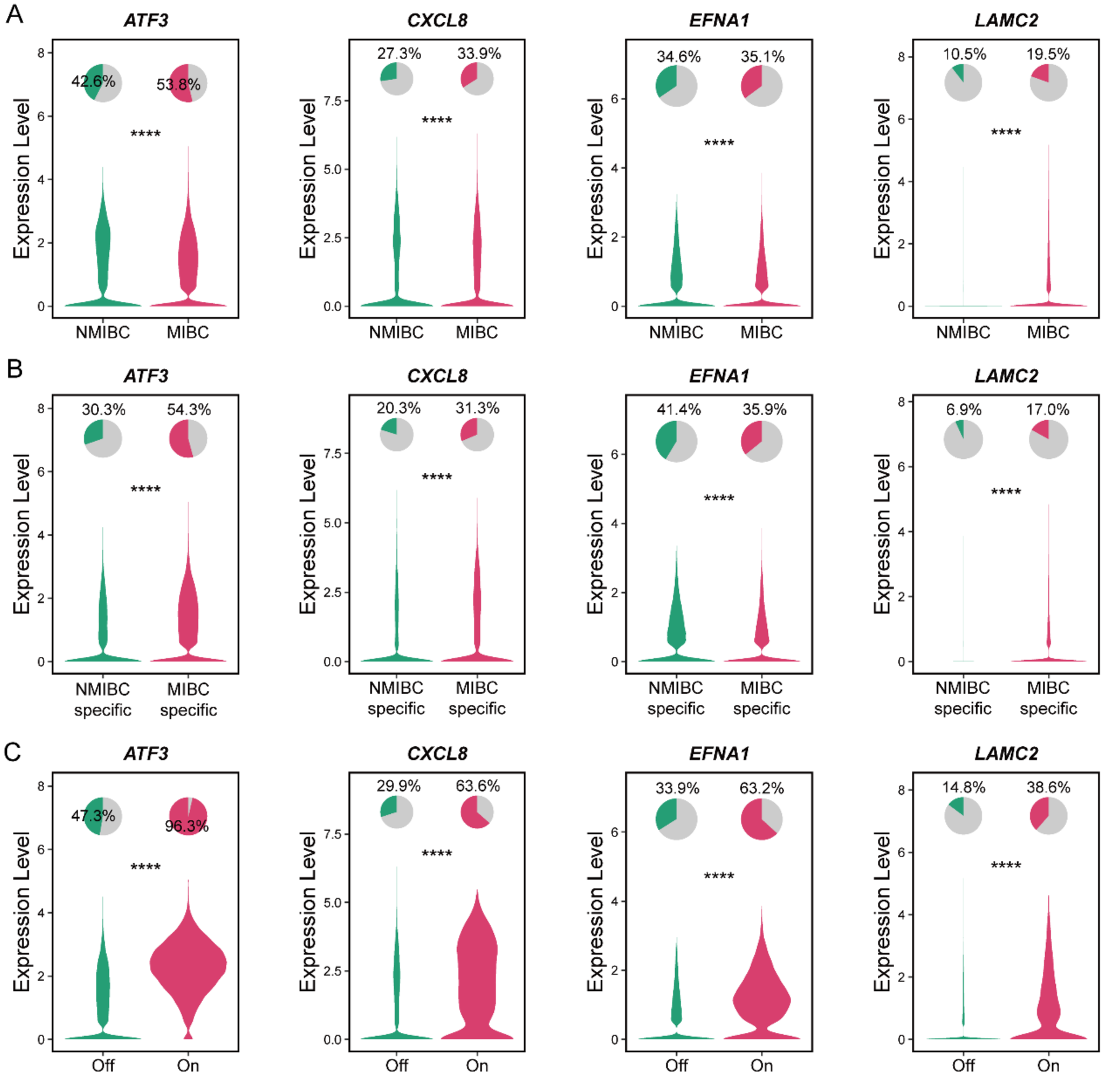

Figure S10. Transcription factor enrichment analysis using "CollecTRI" database, depicting ATF3 as a key regulator among MPS-MP activated cells.

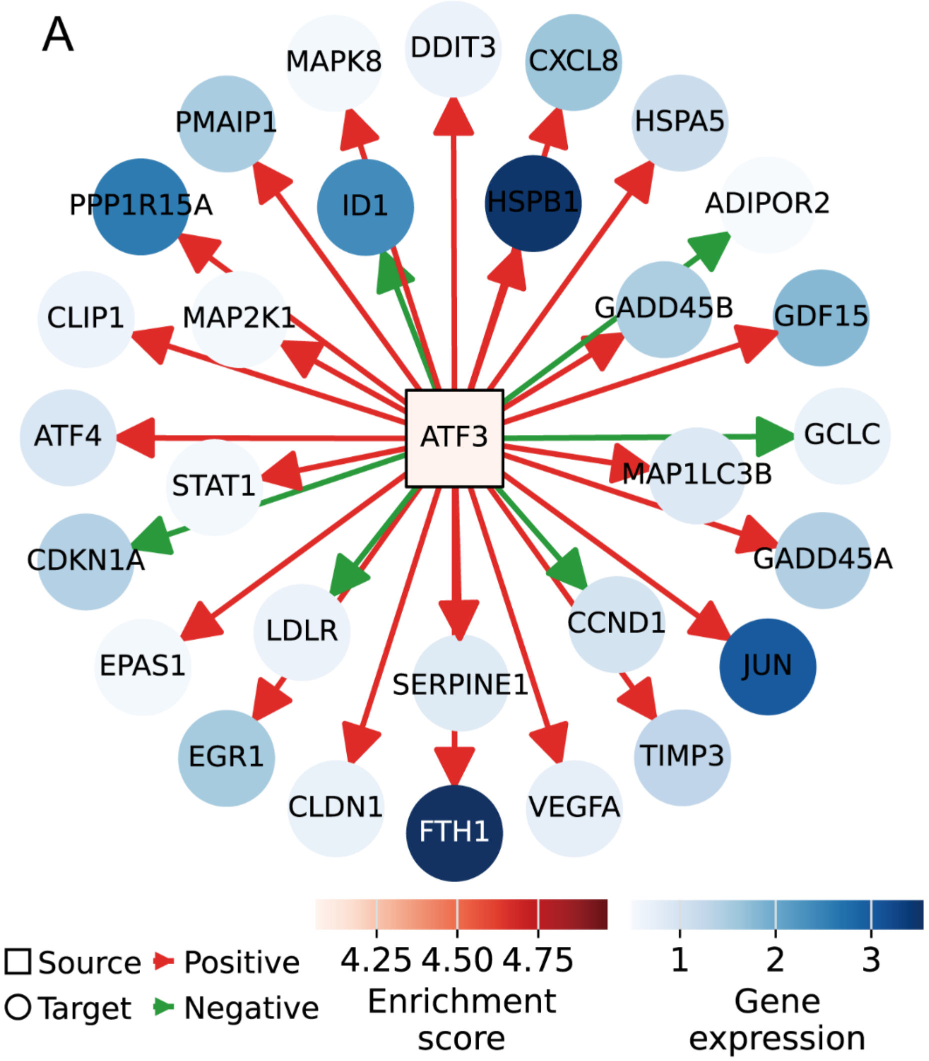

Figure S11. Functional enrichment and pathway annotation of MPS-MP activated cells.

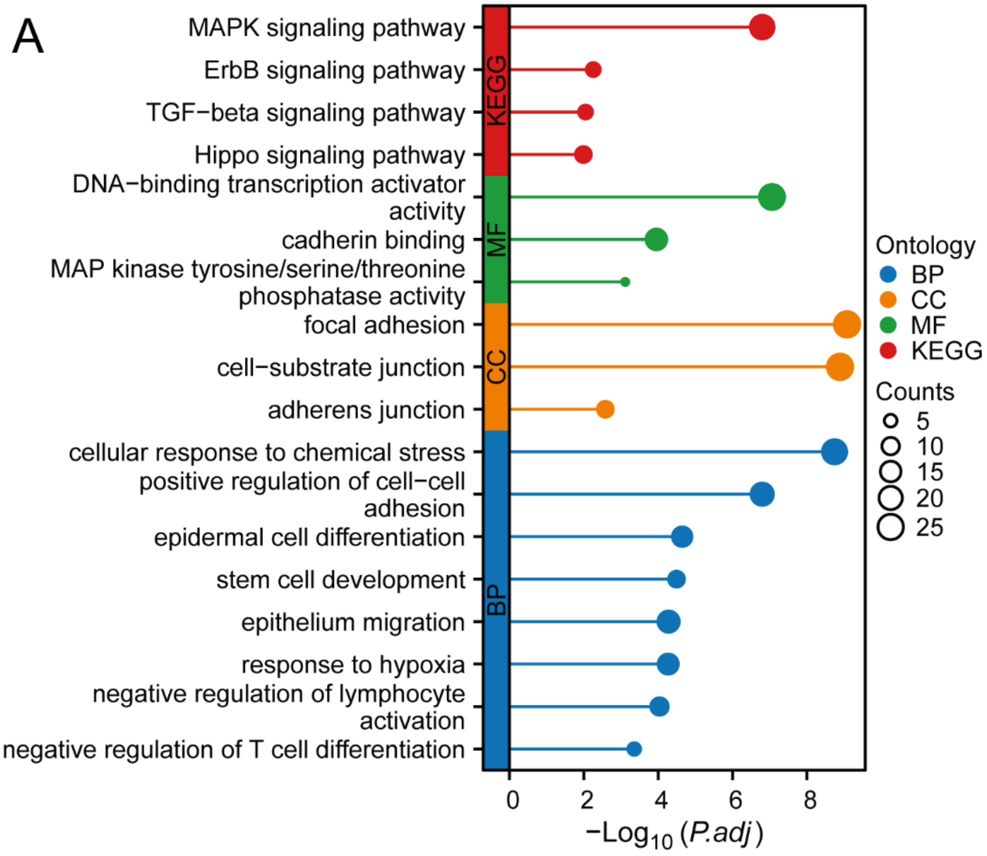

Figure S12. Cell-cell communication analysis between MPS-MP activated/inactivated cells and other stromal and immune cells.

(A) Cell-cell communication analysis between MPS-MP activated and other cells.

(B) Cell-cell communication analysis between MPS-MP inactivated and other cells.

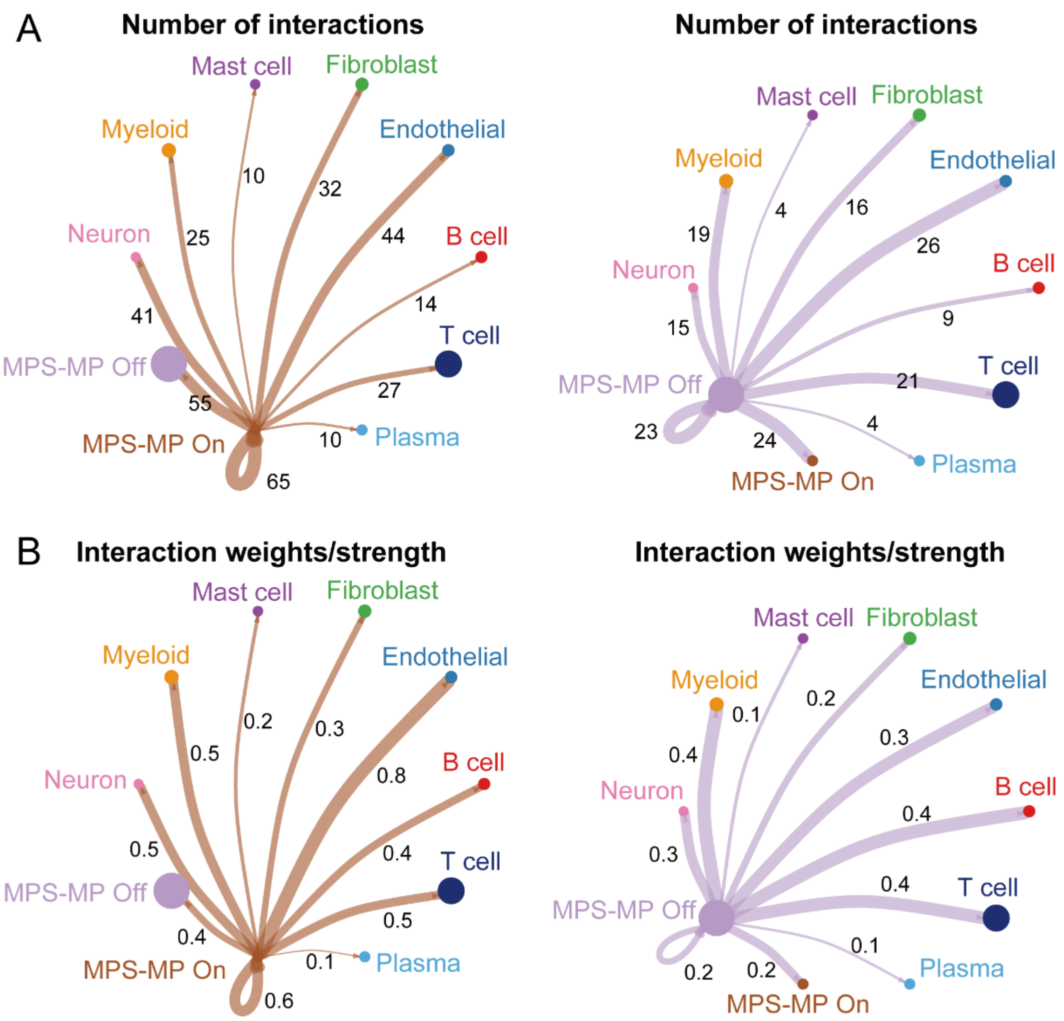

Figure S13. "scSurv" framework identifies survival-associated cellular programs to clinical survival risk.

(A) Schematic overview of the scSurv workflow.

(B) UMAP plot of "scSurv" Z-score contribution to hazard.

(C) Violin plots showing the distribution of "scSurv" Z-score expression levels across major cell populations.

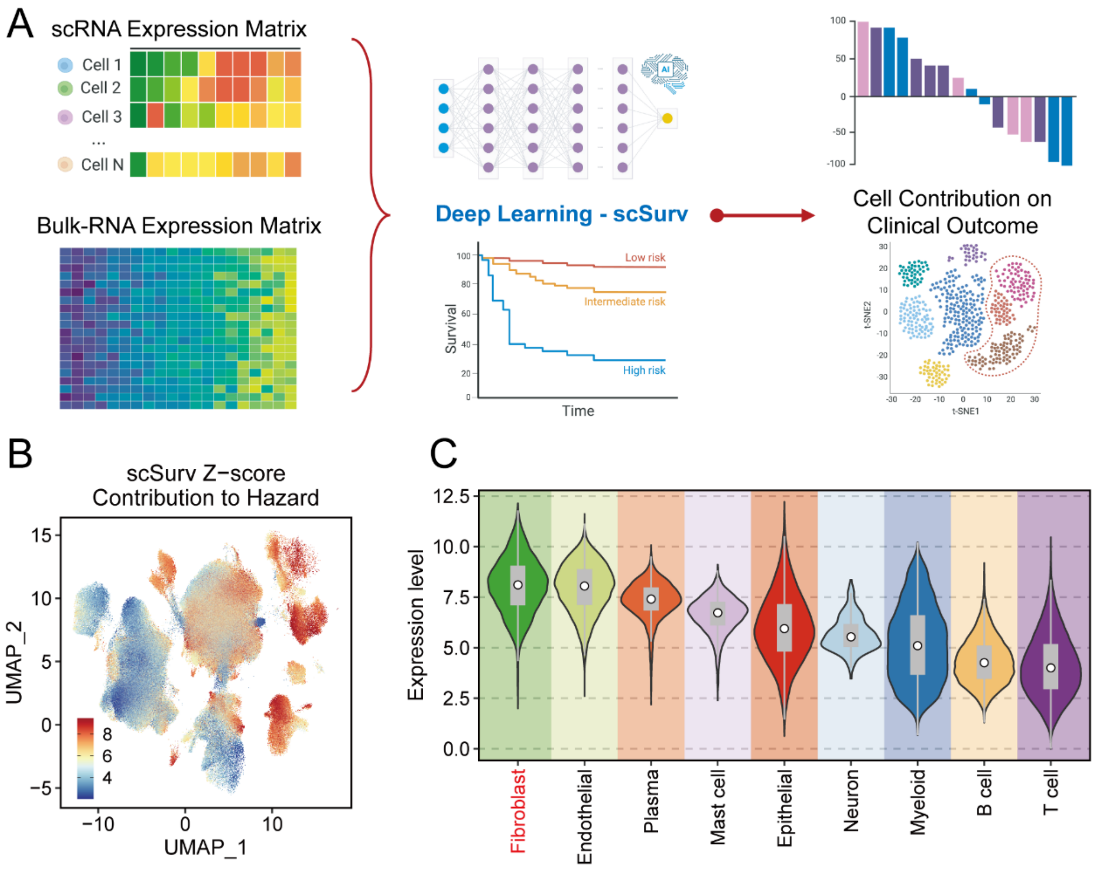

Figure S14. Single-cell mapping of TCGA-derived survival-associated signatures across major cell populations in the tumor microenvironment.

(A–C) UMAP plots showing the distribution of cells colored by module scores for the TCGA overall survival (OS) signature (A), disease-specific survival (DSS) signature (B), and progression-free interval (PFI) signature (C).

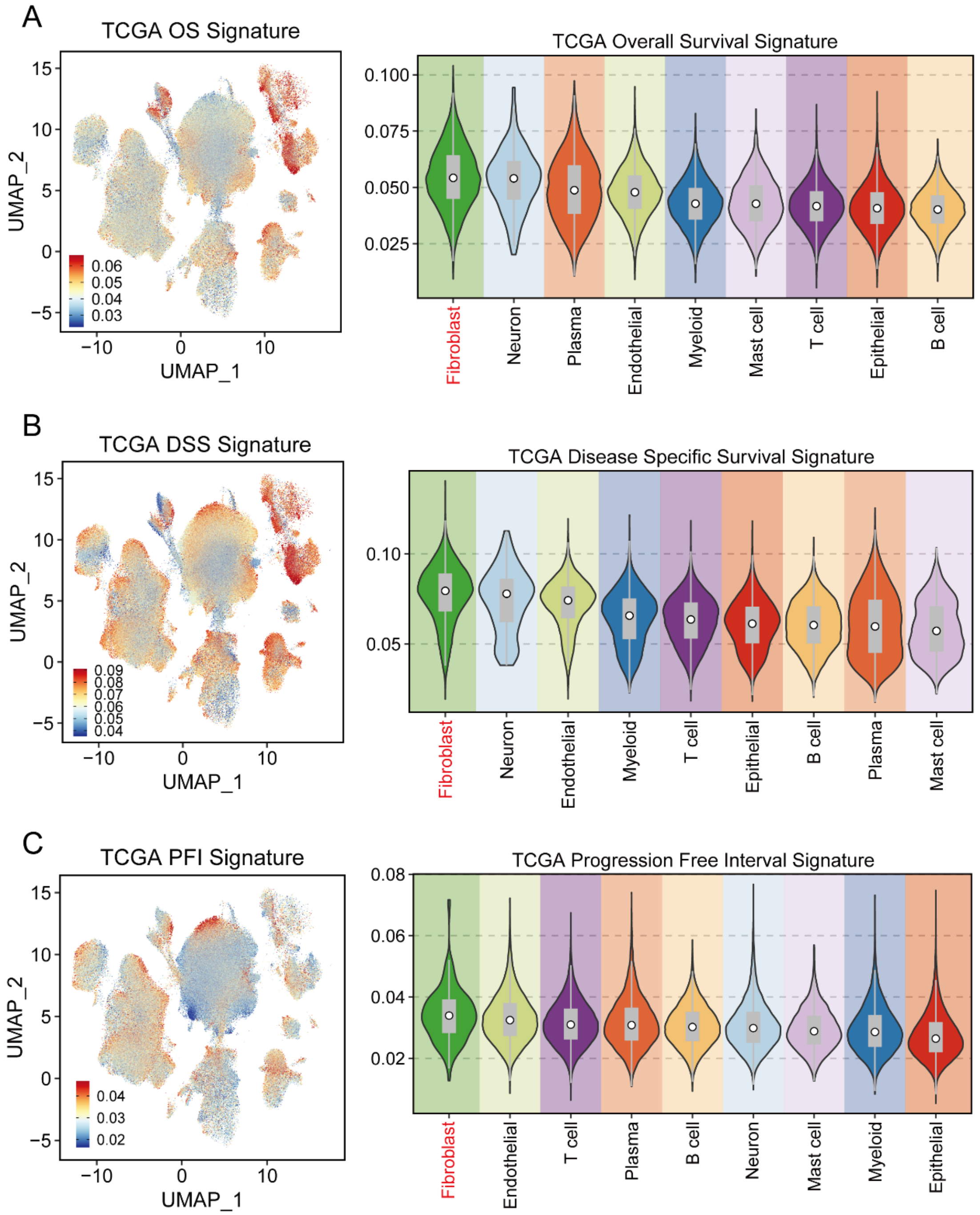

Figure S15. Scissor analysis identifies cell populations associated with multiple survival endpoints in bladder cancer.

(A–C) UMAP plots showing the distribution of Scissor^{+}, Scissor^{-}, and background cells across the single-cell atlas, based on overall survival (OS) (A), disease-specific survival (DSS) (B), and progression-free interval (PFI) (C) phenotypes.

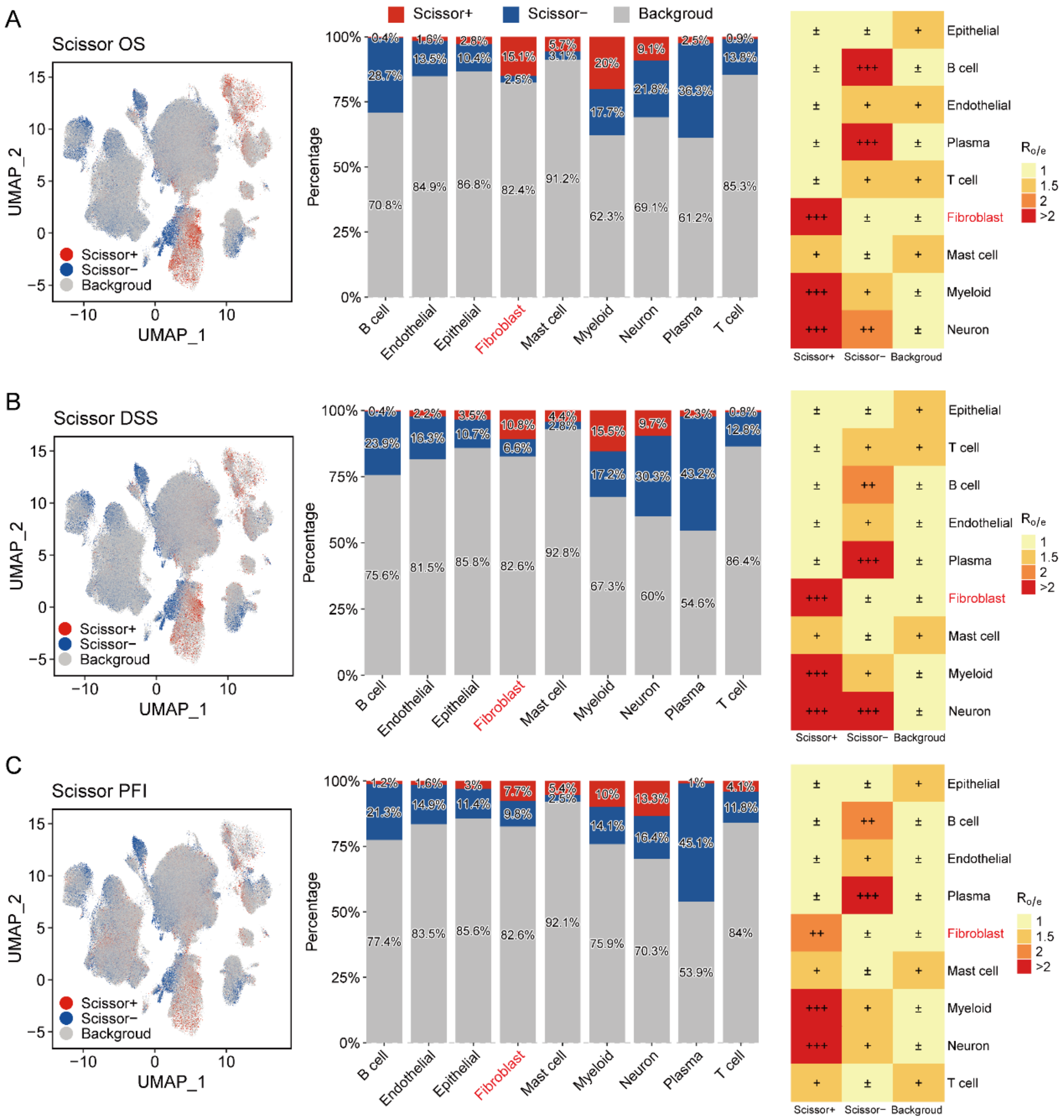

Figure S16. Elevated CAF-related stromal signatures are consistently associated with unfavorable survival in bladder cancer across independent cohorts.

(A) Kaplan-Meier survival curves for patients in the TCGA bladder cancer cohort, stratified into high and low groups according to EPIC (CAFs), MCPcounter (Fibroblasts), and ESTIMATE (Stromal Score) score.

(B) Validation analysis in the Meta-GEO bladder cancer cohort.

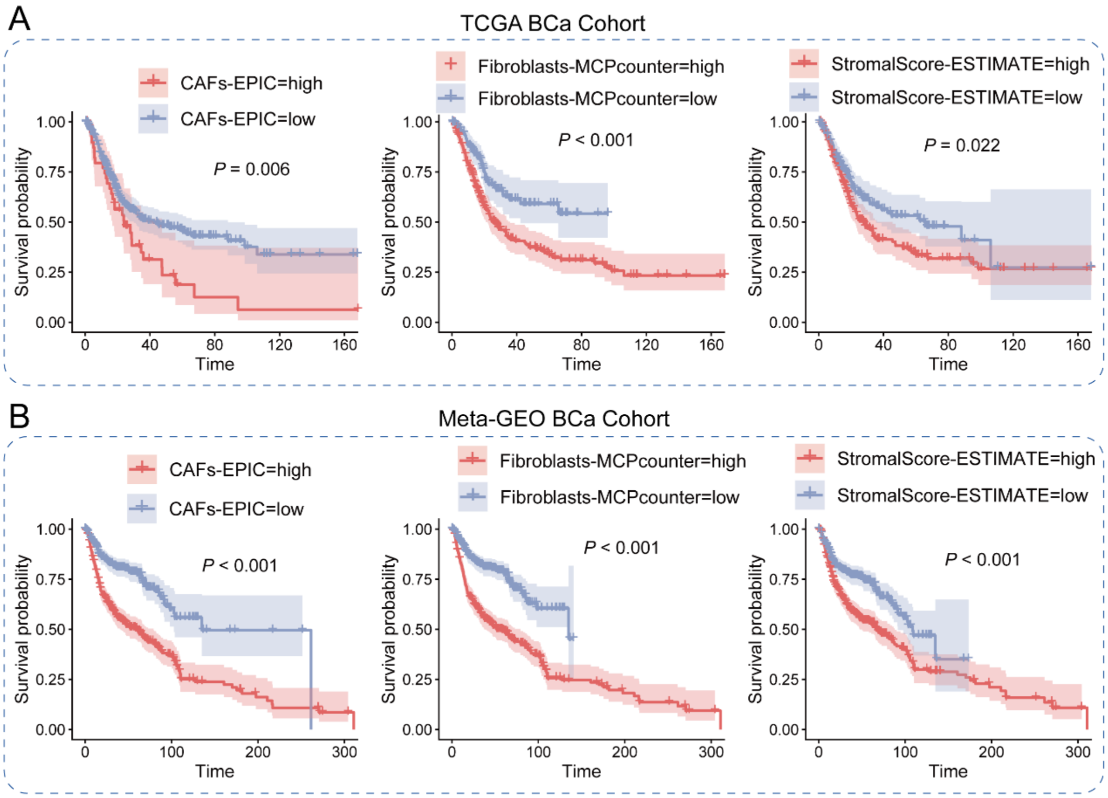

Figure S17. Comprehensive intercellular communication analysis of bladder cancer TME.

(A) Bar plots summarizing the overall cell–cell communication landscape inferred from bladder cancer scRNA-seq atlas.

(B) Differential interaction heatmaps showing changes in the number of interactions and interaction strength among major cell populations.

(C) Comparison of signaling pathway-specific information flow between normal and tumor tissues.

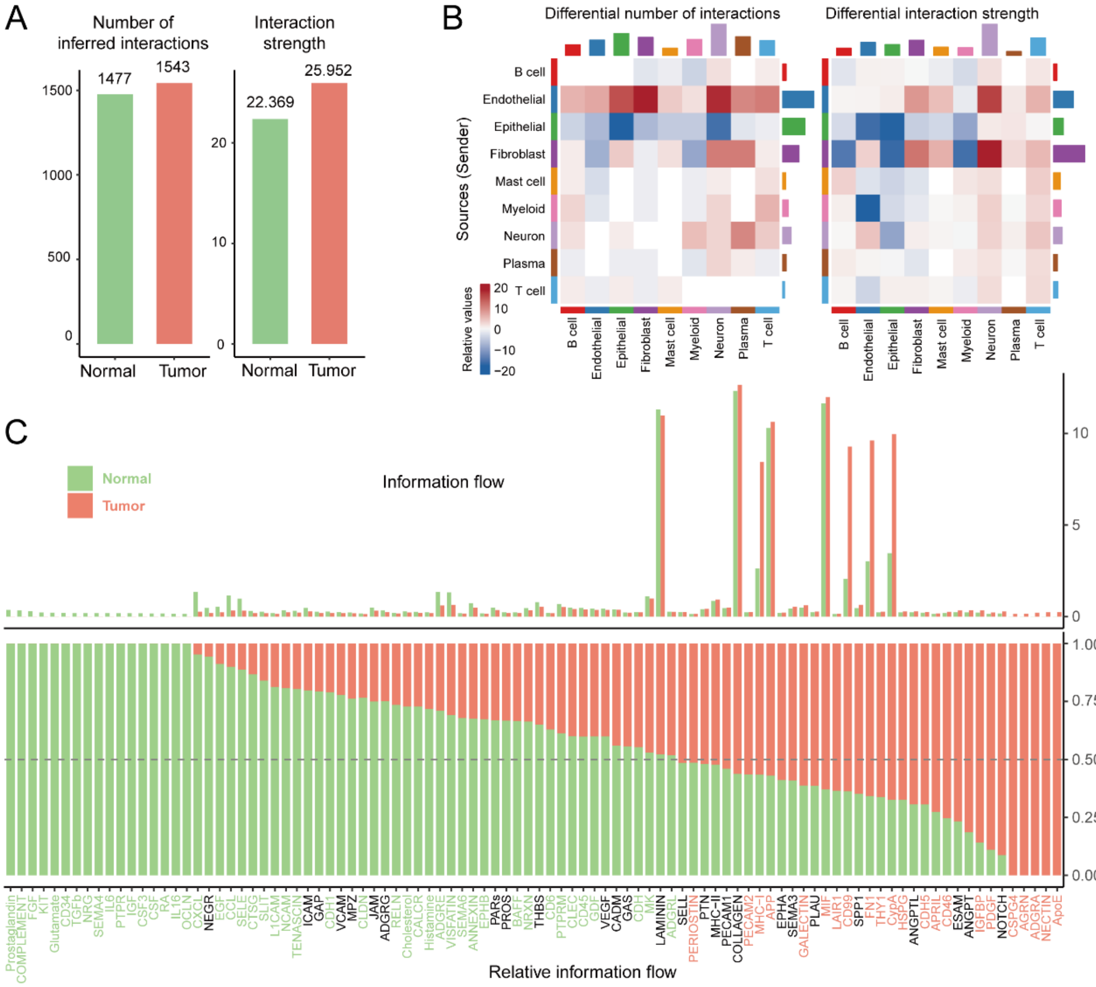

Figure S18. Single-cell transcriptomic landscape of fibroblast populations in bladder cancer.

(A) UMAP visualization of fibroblast-lineage cells identified from human bladder samples.

(B) UMAPs colored by sample origin showing the distribution of fibroblast-lineage cells.

(C) Violin plots of pan-CAF markers across fibroblast clusters, together with dot plots of subtype-defining genes expression level.

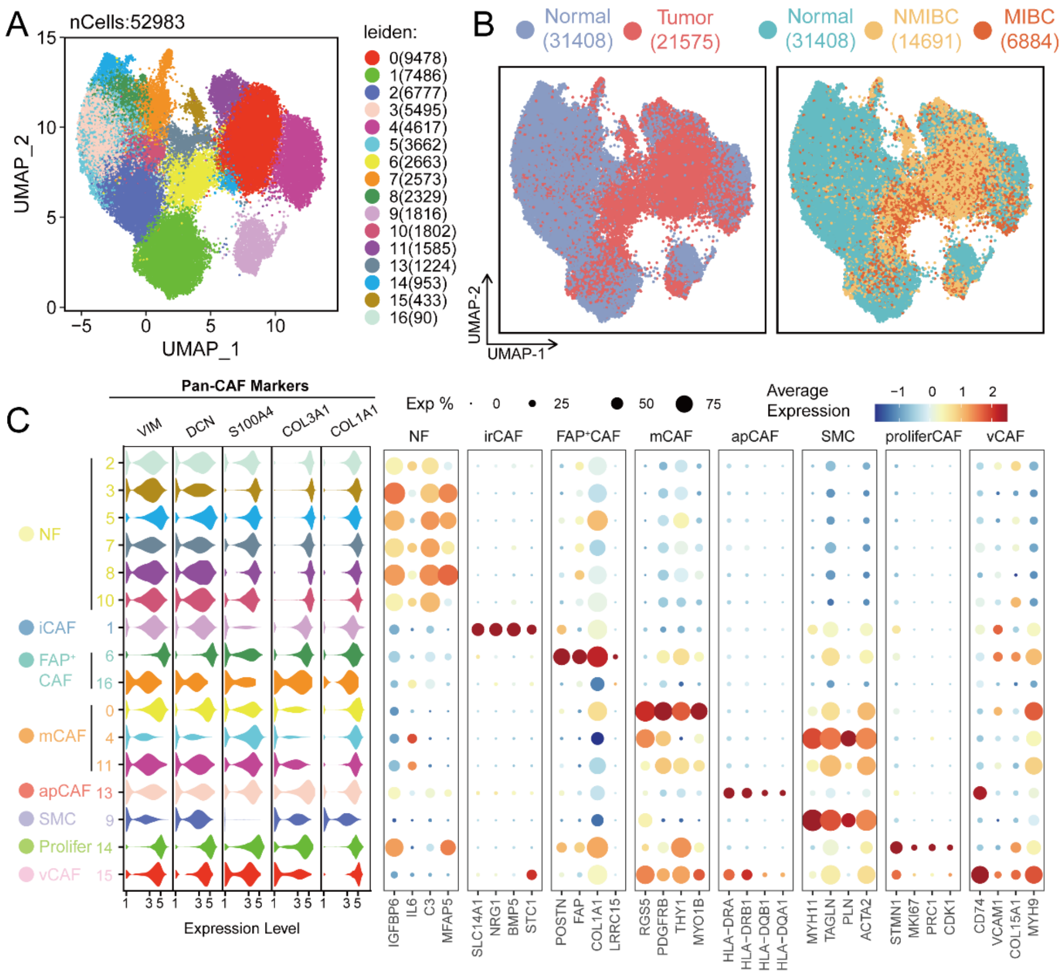

Figure S19. Disease stage dependent remodeling of CAF subpopulations in bladder cancer.

(A) UMAP projection of fibroblast-lineage cells stratified by tissue source.

(B) Stacked bar plot showing the relative proportions of fibroblast subpopulations across different tissues.

(C) Differential abundance analysis comparing tumor versus normal tissues.

(D) Differential abundance analysis comparing MIBC with NMIBC.

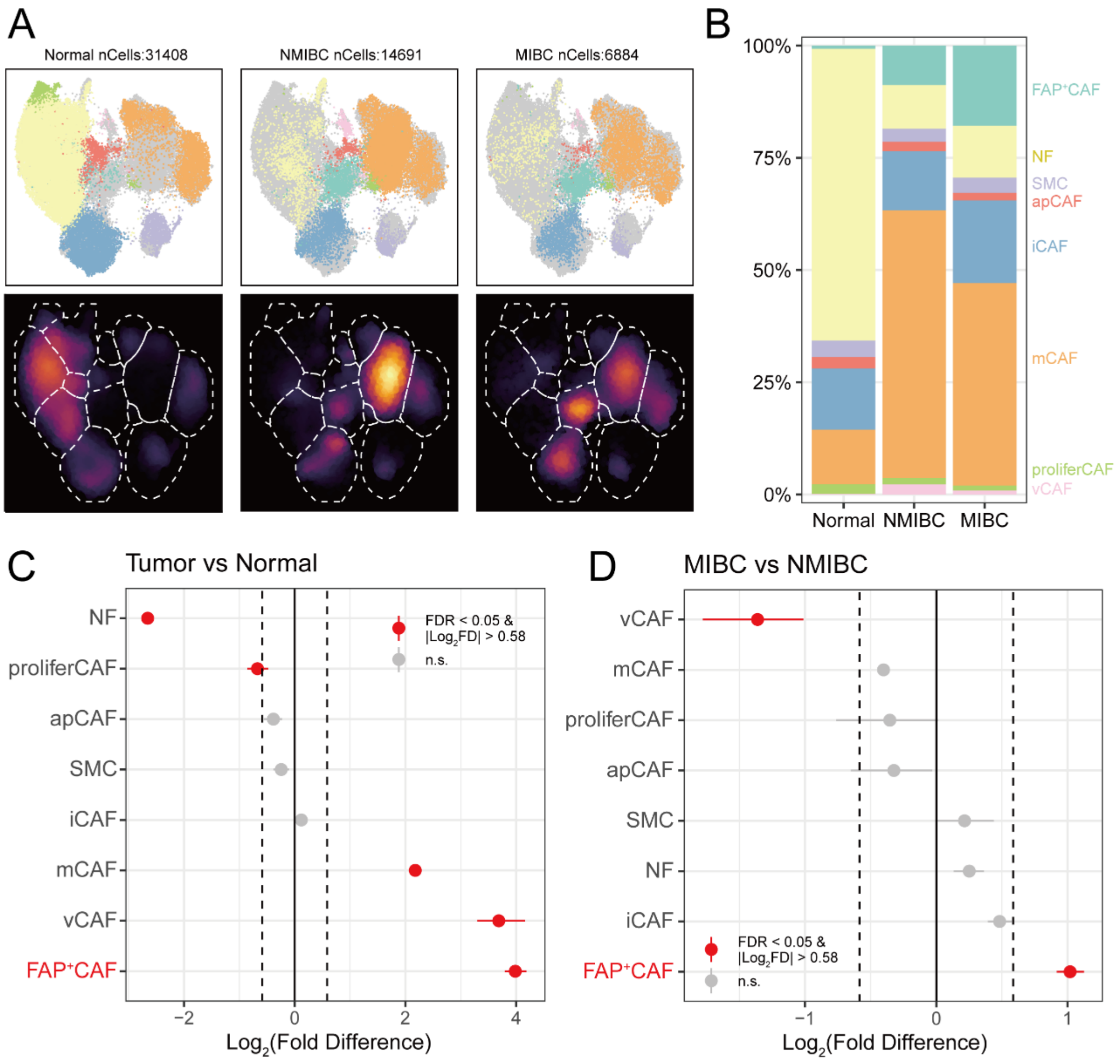

Figure S20. Prognostic-related signatures are differentially enriched across CAF subpopulations.

(A–C) UMAP and violin plots showing the distribution of TCGA-derived survival-associated signature scores across CAF cells, including OS (A), DSS (B), and PFI (C).

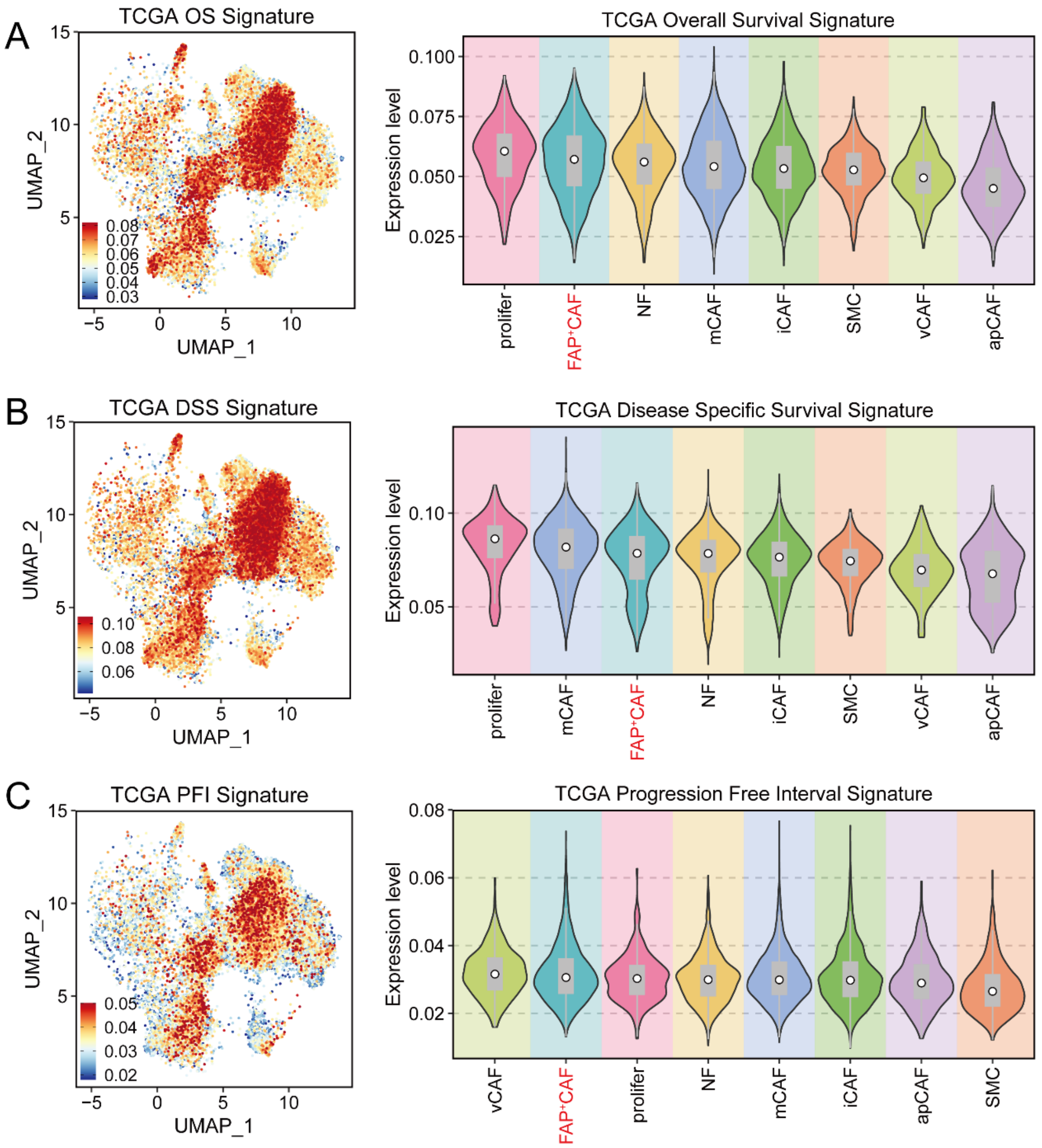

Figure S21. Scissor analysis links distinct CAF states to survival outcomes in bladder cancer.

(A–C) Integrated CAF cells overlaid with Scissor labels derived from TCGA bladder cancer cohort for OS (A), DSS (B), and PFI (C).

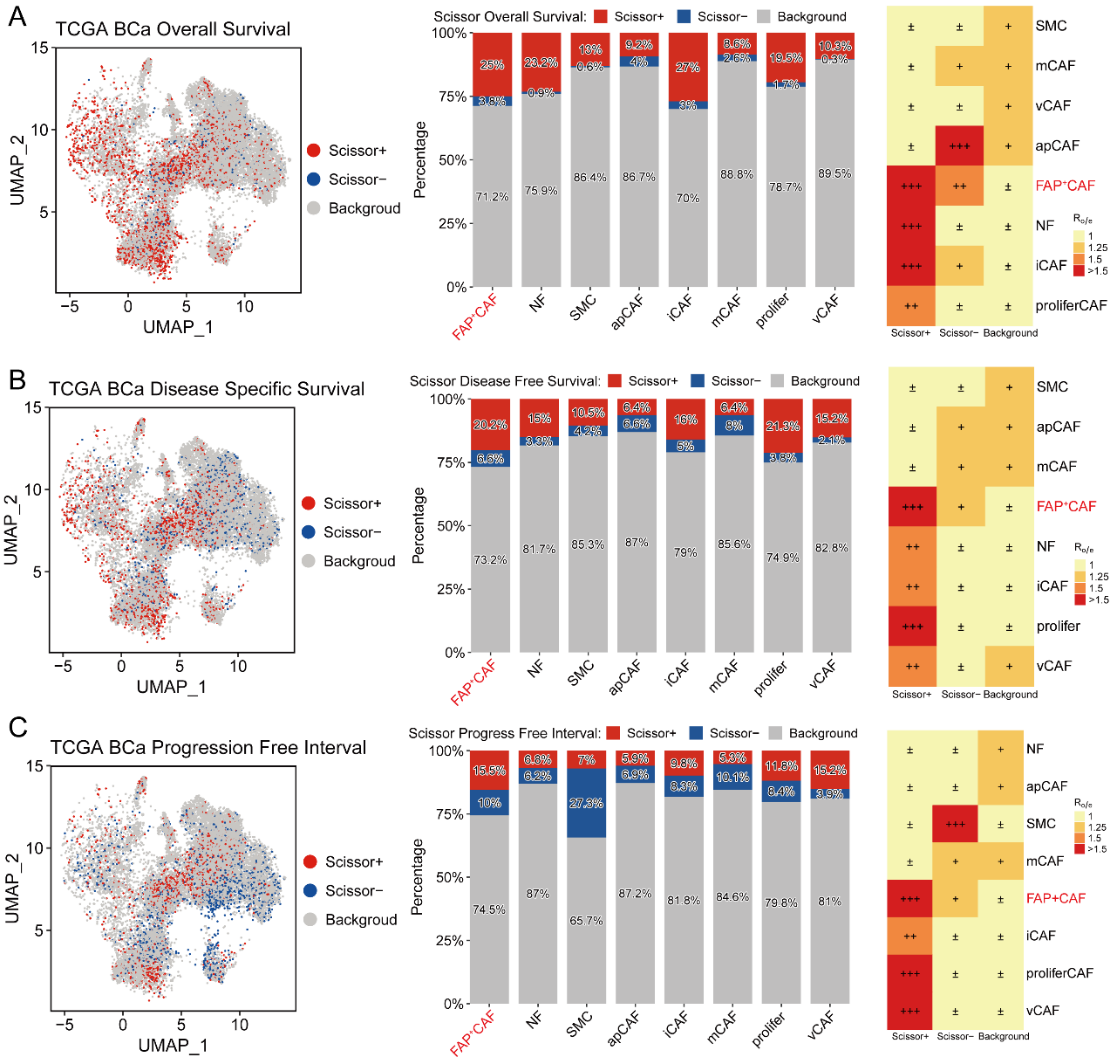

Figure S22. Functional characterization of CAF subpopulations based on "CancerSEA" and "Hallmark" gene-set enrichment analyses.

(A) Heatmap showing the relative activity of "CancerSEA"-defined functional states across CAF cells.

(B) Heatmap depicting the enrichment of MSigDB "Hallmark" pathways in the CAF subclusters.

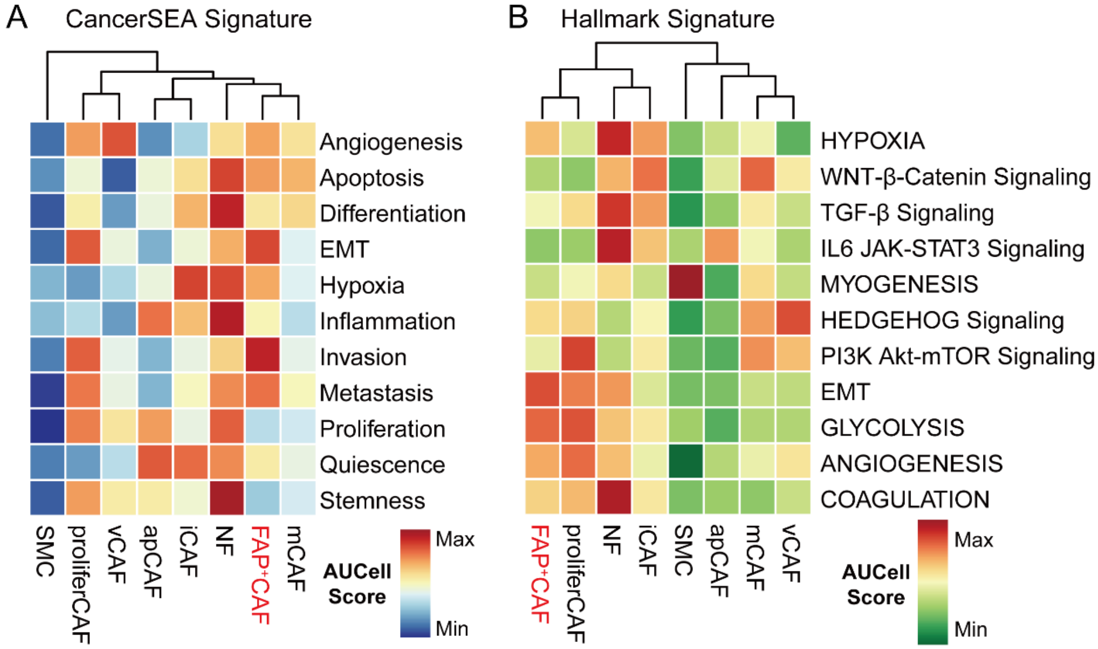

Figure S23. Kaplan-Meier survival analyses in the merged GEO bladder cancer cohort stratified by FAP$^+$ CAFs enrichment level.

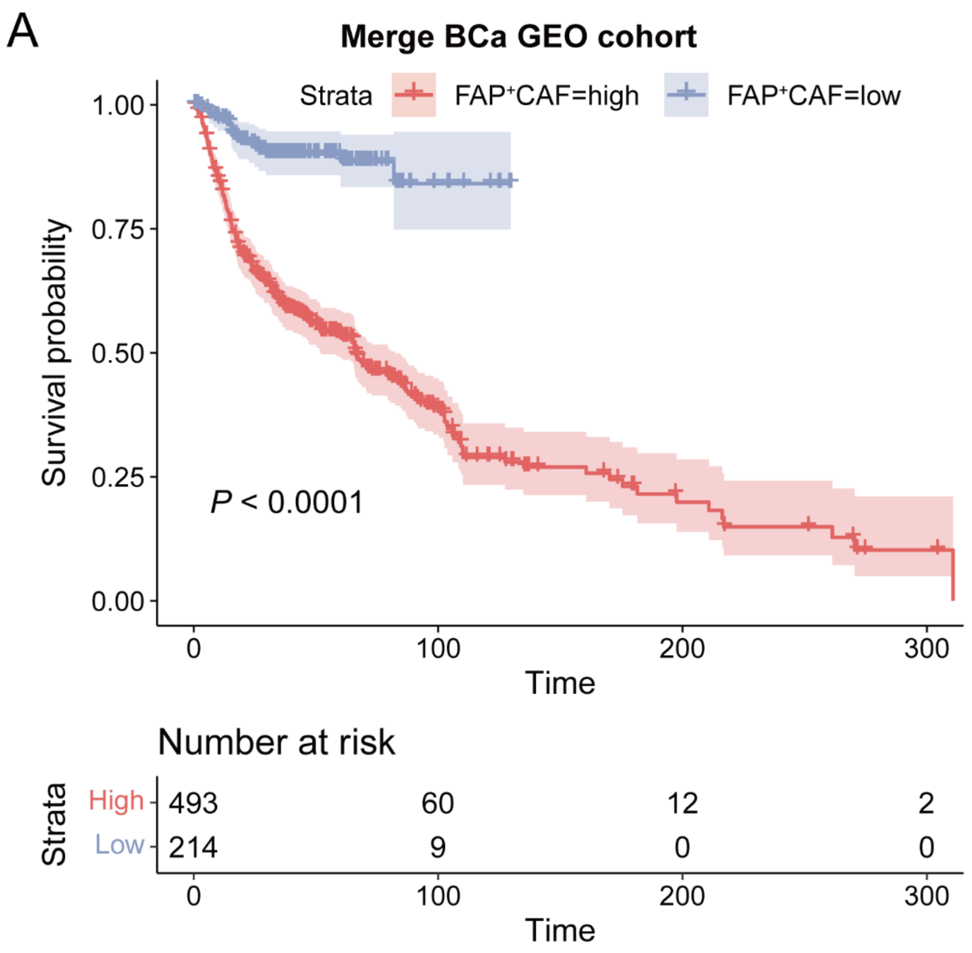

Figure S24. Elevated FAP expression in tumors is associated with poor clinical outcomes.

(A) Comparison of FAP expression between normal and tumor tissues.

(B) Paired analysis of FAP expression in matched normal and tumor tissues.

(C–E) Kaplan–Meier survival analyses stratified by FAP expression level for OS (C), DSS (D), and PFI (E).

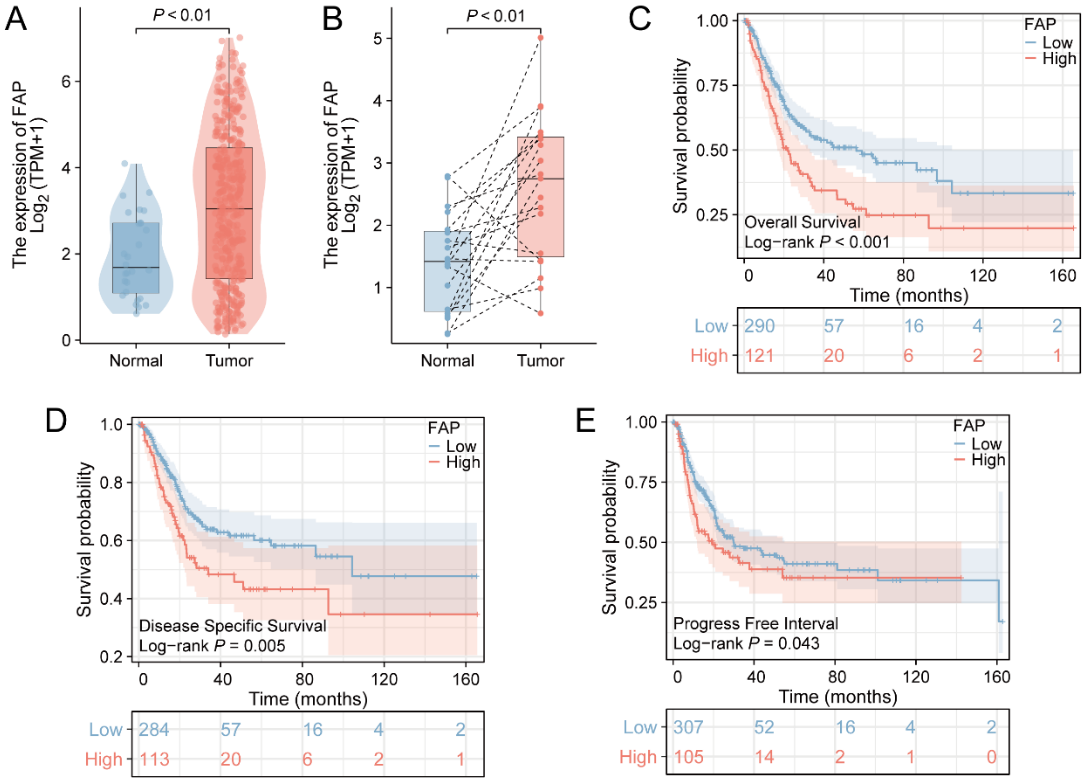

Figure S25. Violin plots showing the distribution of bladder cancer scDRS score across MPS-MP activated/inactivated and fibroblast cell subpopulations.

*: $P < 0.05$

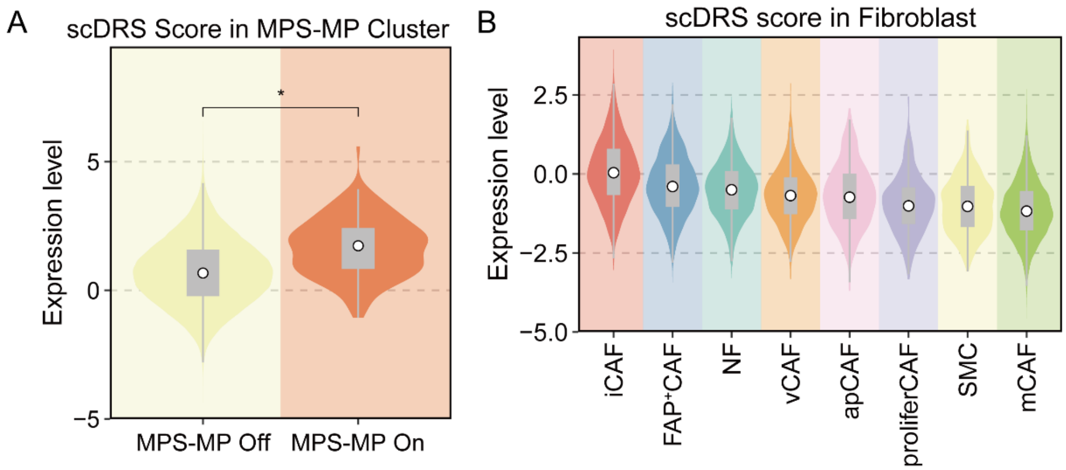

Figure S26. Quality assessment of spatial transcriptomic data in bladder cancer tumor specimens.

(A) Representative spatial maps showing the distribution of RNA counts (nCount_RNA) and gene counts (nFeature_RNA) in two bladder cancer tumor sections.

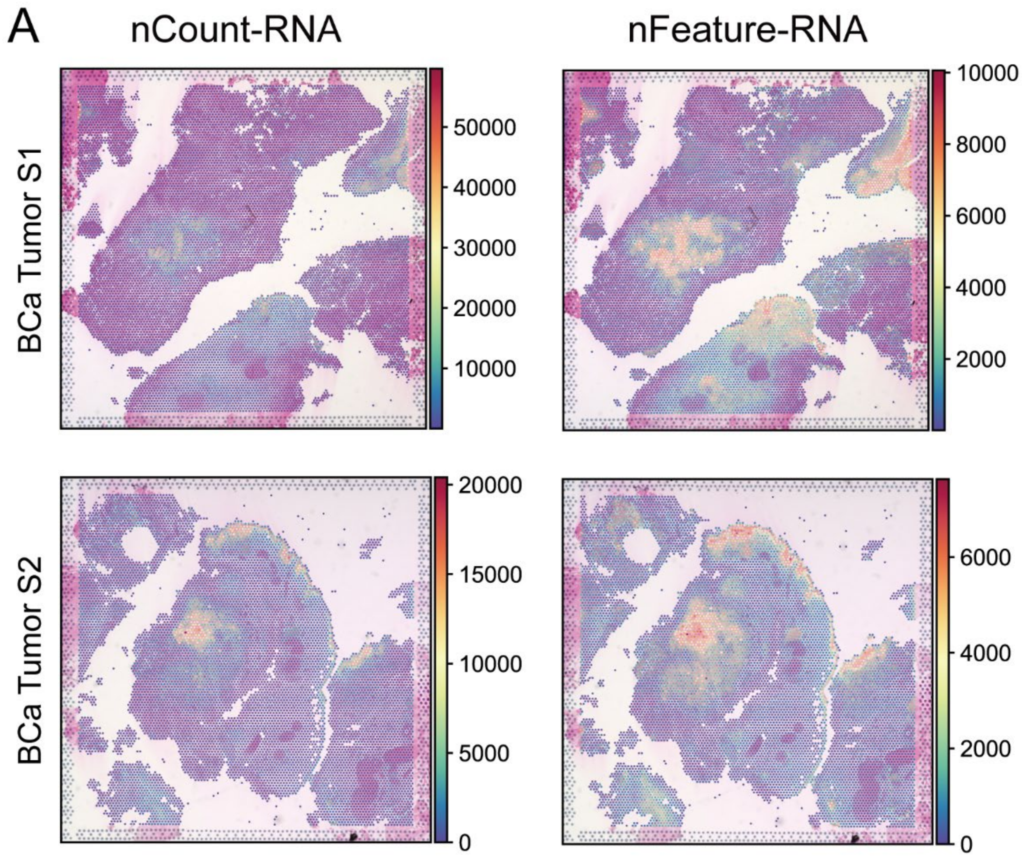

Figure S27. Spatial distribution and correlation analysis of FAP$^+$ CAFs and malignant epithelial tumor cells.

(A) Spatial distribution of FAP$^+$ CAFs and malignant tumor cells in sample S2.

(B) Correlation analysis between FAP$^+$ CAFs and malignant tumor cells in sample S1.

(C) Correlation analysis between FAP$^+$ CAFs and malignant tumor cells in sample S2.

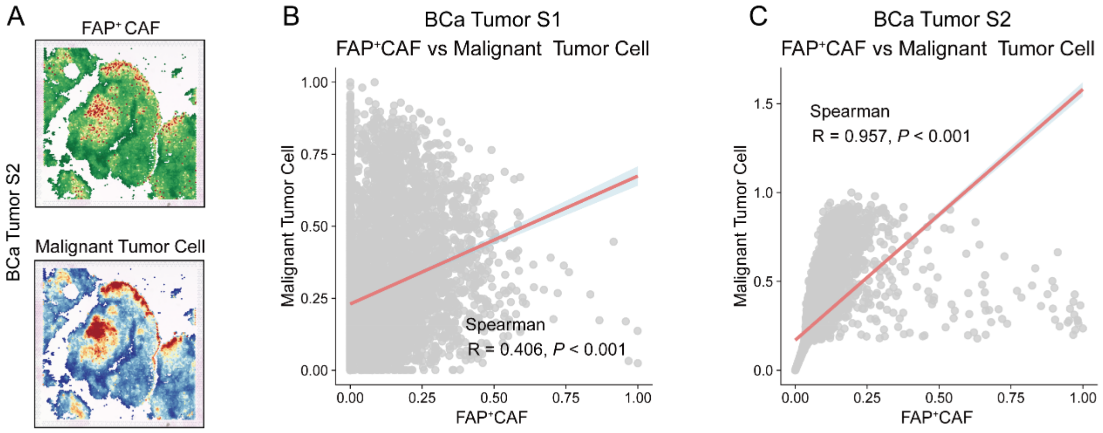

Figure S28. Immunohistochemical staining of POSTN, and COL1A1 in adjacent normal bladder tissue and NMIBC/MIBC bladder cancer tissue. Scale bars = 100 µm.

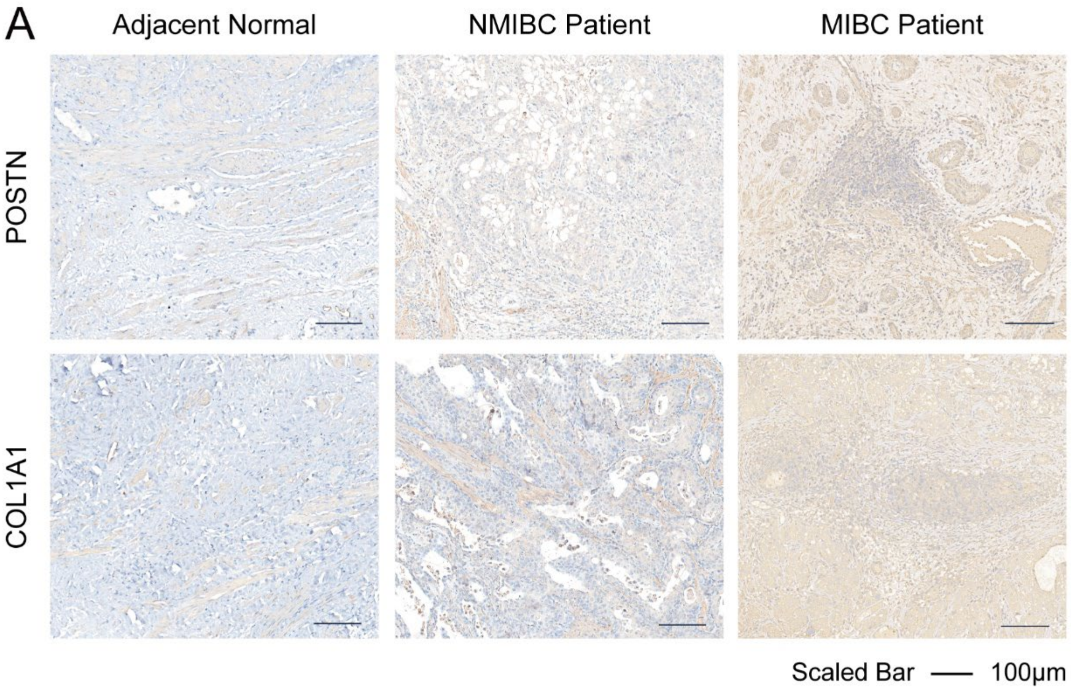

Figure S29. Flow-cytometric isolation and phenotypic validation of FAP⁺ CAFs.

(A) Representative flow-cytometry gating strategy for the purification and quantification of FAP⁺ CAFs from tumor-derived cell suspensions.

(B) Immunofluorescence characterization of isolated NFs and FAP⁺ CAFs.

Scale bar, 100 μm.

*: $P < 0.05$

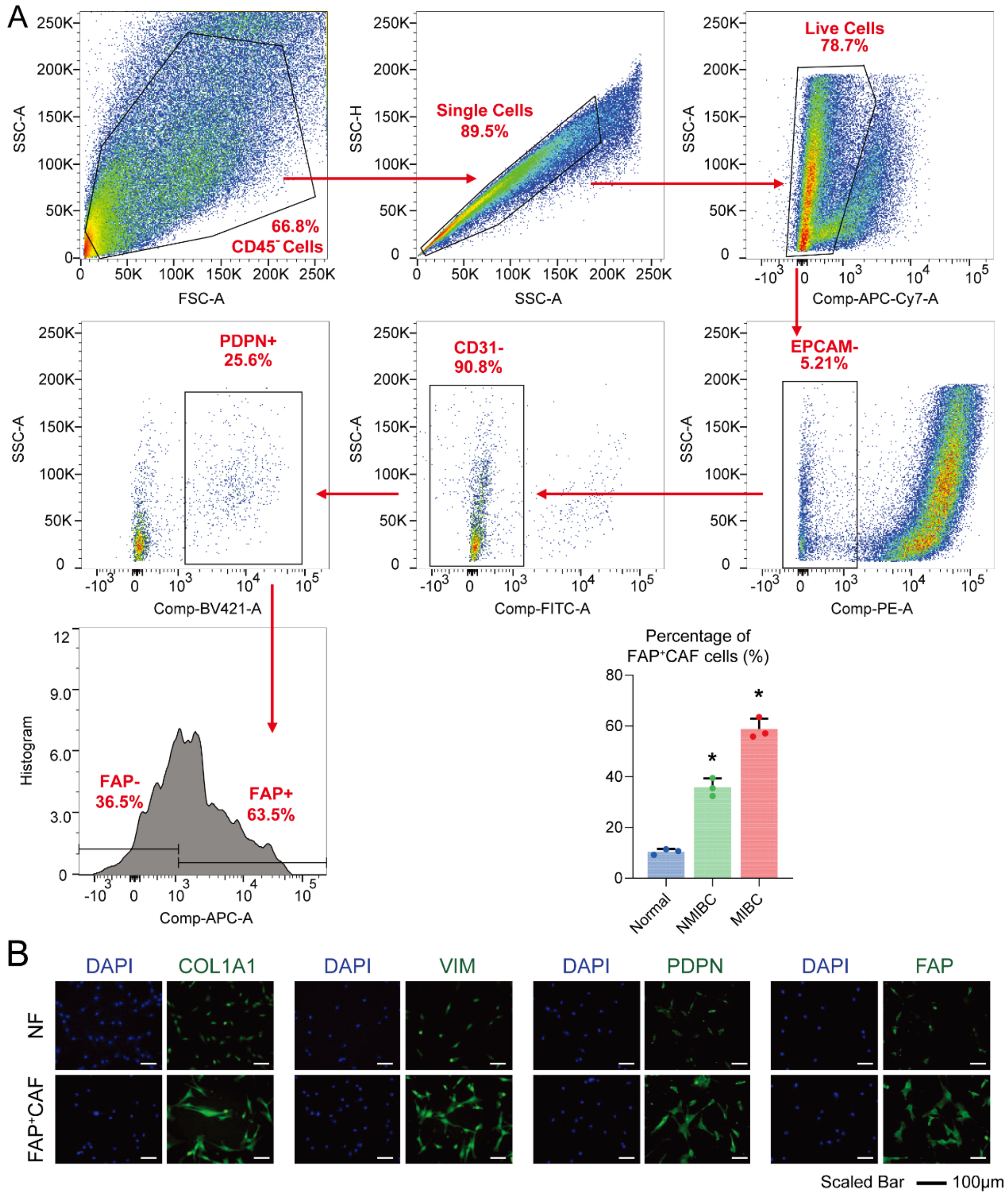

Figure S30. Validation of FAP shRNA knockdown efficiency in primary FAP+ CAF.

*: $P < 0.05$

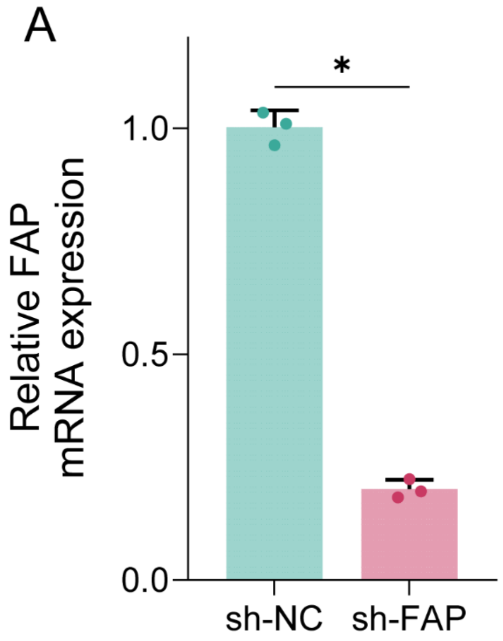

Supplementary Tables

Table S1. Clinicopathological characteristics for Nanjing bladder cancer cohort.

Sample	Age	Gender	Invasive	Stage
BCa01-Normal	80	Male	\	\
BCa01-Tumor1			MIBC	T2N0M0
BCa01-Tumor2				
BCa01-Tumor3				
BCa02-Normal	77	Male	\	\
BCa02-Tumor1			MIBC	T2N0M0
BCa02-Tumor2				
BCa03-Tumor	64	Male	NMIBC	T1N0M0
BCa04-Tumor	65	Male	NMIBC	T1N0M0
BCa05-Tumor	63	Male	MIBC	T3N2M0
BCa06-Tumor	83	Male	MIBC	T3N3M0

Table S2. Public datasets used in this research.

Dataset	Database type
CNP0000460	Single-cell RNA-sequencing
HRA003620	Single-cell RNA-sequencing
PRJNA662018	Single-cell RNA-sequencing
GSE135337	Single-cell RNA-sequencing
GSE217956	Single-cell RNA-sequencing
GSE211388	Single-cell RNA-sequencing
PRJNA609439	Single-cell RNA-sequencing
GSE222315	Single-cell RNA-sequencing
GSE130001	Single-cell RNA-sequencing
GSE192575	Single-cell RNA-sequencing
Salome's cohort	Single-cell RNA-sequencing
HCA cohort	Single-cell RNA-sequencing
GSE129845	Single-cell RNA-sequencing
GSE159929	Single-cell RNA-sequencing
TCGA-BLCA	Bulk RNA-sequencing
GSE13507	Bulk RNA-sequencing
GSE31684	Bulk RNA-sequencing
GSE32894	Bulk RNA-sequencing
GSE48075	Bulk RNA-sequencing
GSE48276	Bulk RNA-sequencing
GSE69795	Bulk RNA-sequencing
GSE70691	Bulk RNA-sequencing
GSE246011	Spatial transcriptomic

Table S3. Differently expressed genes in MPS-MP activated cells compared with MPS-MP inactivated cells.

Gene	Average Log_2FC	PCT-1[#]	PCT-2[#]	*P*-value
JUN	2.026	0.991	0.641	< 0.001
ZFAND2A	1.836	0.675	0.294	< 0.001
TIMP3	1.819	0.632	0.296	< 0.001
PPP1R15A	1.732	0.989	0.590	< 0.001
DUSP2	1.702	0.688	0.280	< 0.001
ATF3	1.687	0.963	0.473	< 0.001
IER3	1.644	0.882	0.506	< 0.001
HSPA6	1.613	0.544	0.227	< 0.001
DNAJB1	1.577	0.969	0.613	< 0.001
CXCL8	1.552	0.636	0.299	< 0.001
BAG3	1.540	0.713	0.264	< 0.001
IER2	1.532	0.945	0.592	< 0.001
ID3	1.520	0.839	0.523	< 0.001
HSPA1B	1.486	0.921	0.572	< 0.001
NFKBIA	1.478	0.928	0.601	< 0.001
PMAIP1	1.465	0.846	0.348	< 0.001
GADD45B	1.449	0.824	0.368	< 0.001
SQSTM1	1.448	0.982	0.744	< 0.001
SOD2	1.446	0.765	0.402	< 0.001
BTG2	1.435	0.924	0.565	< 0.001
HES1	1.408	0.939	0.693	< 0.001
ID1	1.376	0.897	0.717	< 0.001
GADD45A	1.352	0.813	0.383	< 0.001
FOSB	1.350	0.943	0.475	< 0.001
SERPINH1	1.333	0.604	0.233	< 0.001
ZFP36	1.316	0.913	0.515	< 0.001
DUSP1	1.279	0.888	0.519	< 0.001
JUNB	1.274	0.976	0.694	< 0.001

HSPH1	1.273	0.836	0.475	< 0.001
EGR1	1.262	0.854	0.370	< 0.001
ANGPTL4	1.237	0.562	0.240	< 0.001
IER5	1.221	0.735	0.330	< 0.001
MIR23AHG	1.215	0.704	0.259	< 0.001
PLAU	1.210	0.526	0.204	< 0.001
CCN1	1.207	0.460	0.139	< 0.001
MCL1	1.200	0.914	0.534	< 0.001
ABHD3	1.197	0.712	0.304	< 0.001
ABTB2	1.191	0.585	0.205	< 0.001
DDIT4	1.189	0.755	0.431	< 0.001
NFKBIZ	1.183	0.676	0.260	< 0.001
CEBPB	1.180	0.825	0.441	< 0.001
ERRFI1	1.169	0.888	0.495	< 0.001
CXCL1	1.168	0.243	0.088	< 0.001
MAFF	1.167	0.851	0.384	< 0.001
RND3	1.165	0.742	0.370	< 0.001
INSIG1	1.164	0.522	0.217	< 0.001
LAMC2	1.164	0.386	0.148	< 0.001
FOS	1.160	0.980	0.713	< 0.001
HMGCS1	1.153	0.668	0.338	< 0.001
FOSL1	1.151	0.793	0.329	< 0.001

[#] PCT-1: Percentage of cells expressing the gene in MPS-MP-On cell subpopulations; PCT-2: Percentage of cells expressing the gene in in MPS-MP-Off cell subpopulations.

Table S4. Oligonucleotide sequences used in this research.

Primers	Sequences (5' → 3')	
FAP	Forward	ATGAGCTTCCTCGTCCAATTCA
	Reverse	AGACCACCAGAGAGCATATTTTG
SOX2	Forward	GCCGAGTGGAAACTTTTGTCG
	Reverse	GGCAGCGTGTACTTATCCTTCT
POU5F1(OCT4)	Forward	AGAGGATCACCTTGGGGTACA
	Reverse	CGAAGCGACAGATGGTGGTC
ALDH1A1	Forward	GCACGCCAGACTTACCTGTC
	Reverse	CCTCCTCAGTTGCAGGATTAAAG
PROM1(CD133)	Forward	AGTCGGAAACTGGCAGATAGC
	Reverse	GGTAGTGTTGTACTGGGCCAAT
CD44	Forward	CTGCCGCTTTGCAGGTGTA
	Reverse	CATTGTGGGCAAGGTGCTATT
β-actin	Forward	ATGACTTAGTTGCGTTACACC
	Reverse	GACTTCCTGTAACAACGCATC
